# Supplementary material for: Synthesis and Evaluation of Antidepressant Activities of 5-Aryl-4,5-dihydrotetrazolo [1,5-a]thieno[2,3-e]pyridine Derivatives
Source: Molecules. 2019 May 14;24(10):1857. doi: 10.3390/molecules24101857 (PMC6572050; doi:10.3390/molecules24101857)

Synthesis and Evaluation of Antidepressant Activities of 5-Aryl-4,5-dihydrotetrazolo[1,5-*a*]thieno[2,3-*e*]pyridine Derivatives

Compounds 1a-p ^{1}H-NMR and ^{13}C-NMR spectral analyze data:

7-(2-Chlorophenyl)-6,7-dihydrothieno[3,2-b]pyridin-5(4H)-one (1a)

Mp: 239-240 °C. ^{1}H-NMR ($CDCl_3$, 300 MHz, ppm) δ: 2.86-3.17 (m, 2H, CH_2), 4.92 (t, 1H, J = 6.7 Hz, CH), 6.76 (d, 1H, J = 5.2 Hz, S-C=C-H), 7.19 (d, 1H, J = 5.2 Hz, S-C-H), 7.07-7.43 (m, 4H, Ar-H), 9.42 (s, 1H, NH). ^{13}C-NMR ($CDCl_3$, 75 MHz) δ: 34.7, 37.7, 116.1, 118.1, 125.2, 127.8, 128.2, 128.9, 129.8, 131.9, 137.8, 139.7, 168.1.

7-(3-Chlorophenyl)-6,7-dihydrothieno[3,2-b]pyridin-5(4H)-one (1b)

Mp: 248-250 °C. ^{1}H-NMR ($CDCl_3$, 300 MHz, ppm) δ: 2.84-3.10 (m, 2H, CH_2), 4.38 (t, 1H, CH), 6.76 (s, 1H, S-C=C-H), 7.16 (s, 1H, S-C-H), 7.16-7.28 (m, 4H, Ar-H), 9.59 (s, 1H, NH). ^{13}C-NMR ($CDCl_3$, 75 MHz) δ: 37.5, 117.5, 118.2, 124.9, 125.8, 126.9, 127.1, 130.6, 133.3, 137.2, 145.5, 168.4.

7-(4-Chlorophenyl)-6,7-dihydrothieno[3,2-b]pyridin-5(4H)-one (1c)

Mp: 237-238 °C. ^{1}H-NMR ($CDCl_3$, 300 MHz, ppm) δ: 2.81-3.08 (m, 2H, CH_2), 4.38 (t, 1H, J = 7.6 Hz, CH), 6.74 (d, 1H, J = 5.3 Hz, S-C=C-H), 7.16 (d, 1H, J = 5.3 Hz, S-C-H), 7.18 (d, 2H, J = 8.5 Hz, Ar-H), 7.33 (d, 2H, J = 8.5 Hz, Ar-H), 9.51 (s, 1H, NH). ^{13}C-NMR ($CDCl_3$, 75 MHz) δ: 37.2, 117.8, 118.2, 124.8, 128.7, 128.7, 128.9, 128.9, 131.7, 137.1, 141.9, 168.4.

7-(2-Fluorophenyl)-6,7-dihydrothieno[3,2-b]pyridin-5(4H)-one (1d)

Mp: 234-235 °C. ^{1}H-NMR ($CDCl_3$, 300 MHz, ppm) δ: 2.88-3.11 (m, 2H, CH_2), 4.76 (t, 1H, J = 6.7 Hz, CH), 6.75 (d, 1H, J = 5.2 Hz, S-C=C-H), 7.17 (d, 1H, J = 5.3 Hz, S-C-H), 7.09-7.27 (m, 4H, Ar-H), 9.36 (s, 1H, NH). ^{13}C-NMR ($CDCl_3$, 75 MHz) δ: 31.3, 37.7, 115.5, 116.3, 118.1, 124.8, 124.9, 128.2, 129.1, 129.4, 137.4, 158.4, 168.2.

7-(3-Fluorophenyl)-6,7-dihydrothieno[3,2-b]pyridin-5(4H)-one (1e)

Mp: 244-245 °C. ^{1}H-NMR ($CDCl_3$, 300 MHz, ppm) δ: 2.84-3.11 (m, 2H, CH_2), 4.40 (t, 1H, J = 7.4 Hz, CH), 6.76 (d, 1H, J = 5.2 Hz, S-C=C-H), 7.16 (d, 1H, J = 5.2 Hz, S-C-H), 6.94-7.35 (m, 4H, Ar-H), 9.82 (s, 1H, NH). ^{13}C-NMR ($CDCl_3$, 75 MHz) δ: 37.5, 113.8, 113.9, 117.7, 118.2, 123.2, 124.8, 130.6, 137.1, 145.8, 161.1, 168.4.

7-(4-Fluorophenyl)-6,7-dihydrothieno[3,2-b]pyridin-5(4H)-one (1f)

Mp: 251-253 °C. ^{1}H-NMR ($CDCl_3$, 300 MHz, ppm) δ: 2.82-3.08 (m, 2H, CH_2), 4.39 (t, 1H, J = 7.7 Hz,

CH), 6.75 (d, 1H, *J* = 5.2 Hz, S-C=C-H), 7.15 (d, 1H, *J* = 5.2 Hz, S-C-H), 7.01-7.25 (m, 4H, Ar-H), 9.49 (s, 1H, NH). ^{13}C-NMR ($CDCl_3$, 75 MHz) δ: 37.1, 115.3, 115.5, 118.1,118.3, 124.7, 128.9, 129.0, 136.9, 139.1, 159.9, 168.5.

7-[2-(Trifluoromethyl)phenyl]-6,7-dihydrothieno[3,2-b]pyridin-5(4H)-one (1g)

Mp: 231-233 ºC. ^{1}H-NMR ($CDCl_3$, 300 MHz, ppm) δ: 2.80-3.17 (m, 2H, CH_2), 4.86 (t, 1H, *J* = 7.6 Hz, CH), 6.77 (d, 1H, *J* = 5.2 Hz, S-C=C-H), 7.17 (d, 1H, *J* = 5.2 Hz, S-C-H), 7.37-7.72 (m, 4H, Ar-H), 9.86 (s, 1H, NH). ^{13}C-NMR ($CDCl_3$, 75 MHz) δ: 34.7, 117.7, 118.7, 123.5, 125.8, 126.2, 126.57, 128.2, 129.8, 133.7, 138.2, 142.4, 168.3.

7-[3-(Trifluoromethyl)phenyl]-6,7-dihydrothieno[3,2-b]pyridin-5(4H)-one (1h)

Mp: 242-244 ºC. ^{1}H-NMR ($CDCl_3$, 300 MHz, ppm) δ: 2.86-3.13 (m, 2H, CH_2), 4.48 (t, 1H, *J* = 7.6 Hz, CH), 6.76 (d, 1H, *J* = 5.3 Hz, S-C=C-H), 7.19 (d, 1H, *J* = 5.2 Hz, S-C-H), 7.46-7.56 (m, 4H, Ar-H), 9.23 (s, 1H, NH). ^{13}C-NMR ($CDCl_3$, 75 MHz) δ: 37.5, 117.4, 118.2, 123.7, 123.9, 125.0, 129.2, 129.5, 129.9, 131.3, 137.3, 144.4, 168.3.

7-[4-(Trifluoromethyl)phenyl]-6,7-dihydrothieno[3,2-b]pyridin-5(4H)-one (1i)

Mp: 248-250 ºC. ^{1}H-NMR ($CDCl_3$, 300 MHz, ppm) δ: 2.85-3.14 (m, 2H, CH_2), 4.47 (t, 1H, *J* = 7.4 Hz, CH), 6.77 (d, 1H, *J* = 5.2 Hz, S-C=C-H), 7.18 (d, 1H, *J* = 5.2 Hz, S-C-H), 7.37 (d, 2H, *J* = 7.9 Hz, Ar-H), 7.62 (d, 2H, *J* = 8.0 Hz, Ar-H), 9.72 (s, 1H, NH). ^{13}C-NMR ($CDCl_3$, 75 MHz) δ: 38.1, 117.7, 118.7, 123.3, 125.5, 125.2, 128.1, 128.5, 128.5, 128.7, 137.8, 148.2, 168.7.

7-(2-Methoxyphenyl)-6,7-dihydrothieno[3,2-b]pyridin-5(4H)-one (1j)

Mp: 229-231 ºC. ^{1}H-NMR ($CDCl_3$, 300 MHz, ppm) δ: 2.88-3.10 (m, 2H, CH_2), 3.88 (s, 1H, OCH_3), 4.80 (t, 1H, *J* = 6.6 Hz, CH), 6.71 (d, 1H, *J* = 5.2 Hz, S-C=C-H), 7.13 (d, 1H, *J* = 5.3 Hz, S-C-H), 6.87-7.25 (m, 4H, Ar-H), 9.01 (s, 1H, NH). ^{13}C-NMR ($CDCl_3$, 75 MHz) δ: 31.4, 37.5, 55.3, 111.1, 117.3, 117.9, 120.5, 124.5, 126.6, 128.3, 130.4, 137.3, 155.9, 168.7.

7-(3-Methoxyphenyl)-6,7-dihydrothieno[3,2-b]pyridin-5(4H)-one (1k)

Mp: 238-240 ºC. ^{1}H-NMR ($CDCl_3$, 300 MHz, ppm) δ: 2.87-3.08 (m, 2H, CH_2), 3.80 (s, 1H, OCH_3), 4.38 (t, 1H, *J* = 7.9 Hz, CH), 6.74 (d, 1H, *J* = 5.3 Hz, S-C=C-H), 7.14 (d, 1H, *J* = 5.3 Hz, S-C-H), 6.82-7.30 (m, 4H, Ar-H), 9.48 (s, 1H, NH). ^{13}C-NMR ($CDCl_3$, 75 MHz) δ: 37.9, 54.9, 112.2, 113.0, 118.1, 118.3, 119.2, 124.6, 129.8, 136.9, 144.5, 159.4, 168.6.

7-(4-Methoxyphenyl)-6,7-dihydrothieno[3,2-b]pyridin-5(4H)-one (1l)

Mp: 234-235 ºC. ^{1}H-NMR ($CDCl_3$, 300 MHz, ppm) δ: 2.84-3.05 (m, 2H, CH_2), 3.82 (s, 1H, OCH_3),

4.36 (t, 1H, *J* = 7.9 Hz, CH), 6.72 (d, 1H, *J* = 5.2 Hz, S-C=C-H), 7.14 (d, 1H, *J* = 5.2 Hz, S-C-H), 6.88 (d, 2H, *J* = 8.7 Hz, Ar-H), 7.21 (d, 2H, *J* = 8.5 Hz, Ar-H), 9.98 (s, 1H, NH). ^{13}C-NMR ($CDCl_3$, 75 MHz) δ: 37.2, 55.0, 114.0, 114.0, 118.1, 119.1, 124.4, 128.1, 128.1, 134.8, 136.7, 158.3, 168.7.

7-(4-Bromophenyl)-6,7-dihydrothieno[3,2-b]pyridin-5(4H)-one (1m)

Mp: 246-248 ºC. ^{1}H-NMR ($CDCl_3$, 300 MHz, ppm) δ: 2.81-3.08 (m, 2H, CH_2), 4.37 (t, 1H, *J* = 7.6 Hz, CH), 6.74 (d, 1H, *J* = 5.2 Hz, S-C=C-H), 7.16 (d, 1H, *J* = 5.1 Hz, S-C-H), 7.12 (d, 2H, *J* = 8.4 Hz, Ar-H), 7.49 (d, 2H, *J* = 8.4 Hz, Ar-H), 9.38 (s, 1H, NH). ^{13}C-NMR ($CDCl_3$, 75 MHz) δ: 37.8, 118.2, 118.6, 120.6, 125.3, 129.8, 132.1, 137.6, 137.6, 142.9, 142.9, 168.9.

7-Phenyl-6,7-dihydrothieno[3,2-b]pyridin-5(4H)-one (1n)

Mp: 235-237 ºC. ^{1}H-NMR ($CDCl_3$, 300 MHz, ppm) δ: 2.88-3.09 (m, 2H, CH_2), 4.41 (t, 1H, *J* = 7.6 Hz, CH), 6.75 (d, 1H, *J* = 5.2 Hz, S-C=C-H), 7.14 (d, 1H, *J* = 5.2 Hz, S-C-H), 7.29-7.39 (m, 5H, Ar-H), 9.44 (s, 1H, NH). ^{13}C-NMR ($CDCl_3$, 75 MHz) δ: 38.4, 118.6, 118.9, 125.1, 127.5, 127.5, 127.6, 129.2, 129.2, 137.5, 143.5, 169.1.

7-(2-Chloro-6-fluorophenyl)-6,7-dihydrothieno[3,2-b]pyridin-5(4H)-one (1o)

Mp: 261-263 ºC. ^{1}H-NMR ($CDCl_3$, 300 MHz, ppm) δ: 2.97-3.20 (m, 2H, CH_2), 5.19 (t, 1H, *J* = 8.4 Hz, CH), 6.72 (d, 1H, *J* = 5.3 Hz, S-C=C-H), 7.23 (d, 1H, *J* = 5.0 Hz, S-C-H), 7.02-7.27 (m, 3H, Ar-H), 9.37 (s, 1H, NH). ^{13}C-NMR ($CDCl_3$, 75 MHz) δ: 32.1, 35.3, 114.8, 115.4, 118.0, 124.4, 125.9, 128.2, 129.9, 133.1, 136.6, 162.5, 167.9.

7-(3,4,5-Trimethoxyphenyl)-6,7-dihydrothieno[3,2-b]pyridin-5(4H)-one (1p)

Mp: 312-314 ºC. ^{1}H-NMR ($CDCl_3$, 300 MHz, ppm) δ: 2.85-3.06 (m, 2H, CH_2), 3.82-3.85 (m, 9H, OCH_3), 4.33 (m, 1H, CH), 6.49 (s, 2H, Ar-H), 6.74 (d, 1H, *J* = 5.3 Hz, S-C=C-H), 7.15 (d, 1H, *J* = 5.3 Hz, S-C-H), 9.48 (s, 1H, NH). ^{13}C-NMR ($CDCl_3$, 75 MHz) δ: 55.8, 59.9, 104.5, 118.1, 124.5, 136.4, 136.9, 138.5, 152.9, 168.9.

Compounds 1a-p and target compounds 4a-p, 5, 6, 7 ^{1}H-NMR and ^{13}C-NMR spectrum:

1) Compounds 1a-p ^{1}H-NMR and ^{13}C-NMR spectrum:

7-(2-Chlorophenyl)-6,7-dihydrothieno[3,2-*b*]pyridin-5(4*H*)-one (**1a**)

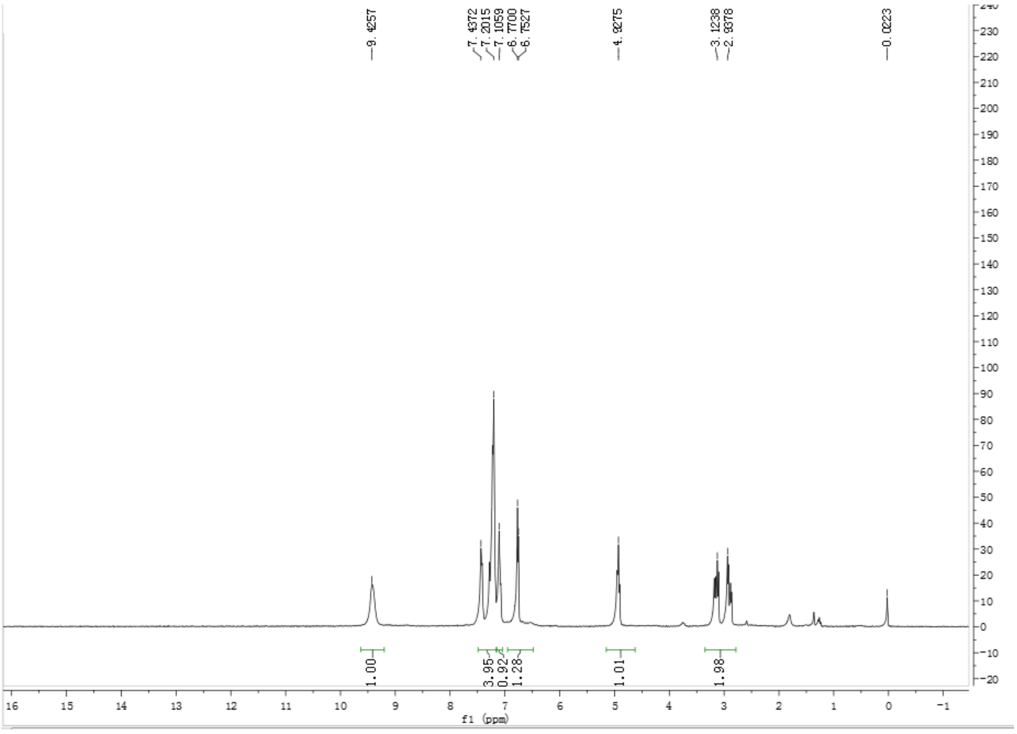

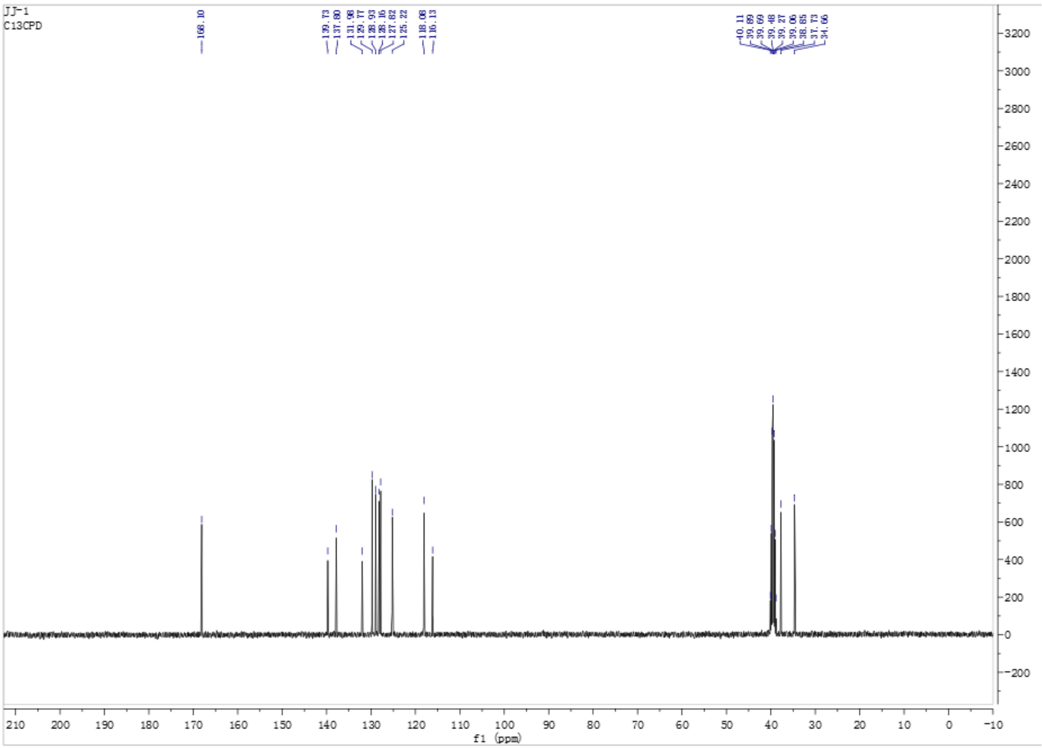

7-(3-Chlorophenyl)-6,7-dihydrothieno[3,2-*b*]pyridin-5(4*H*)-one (**1b**)

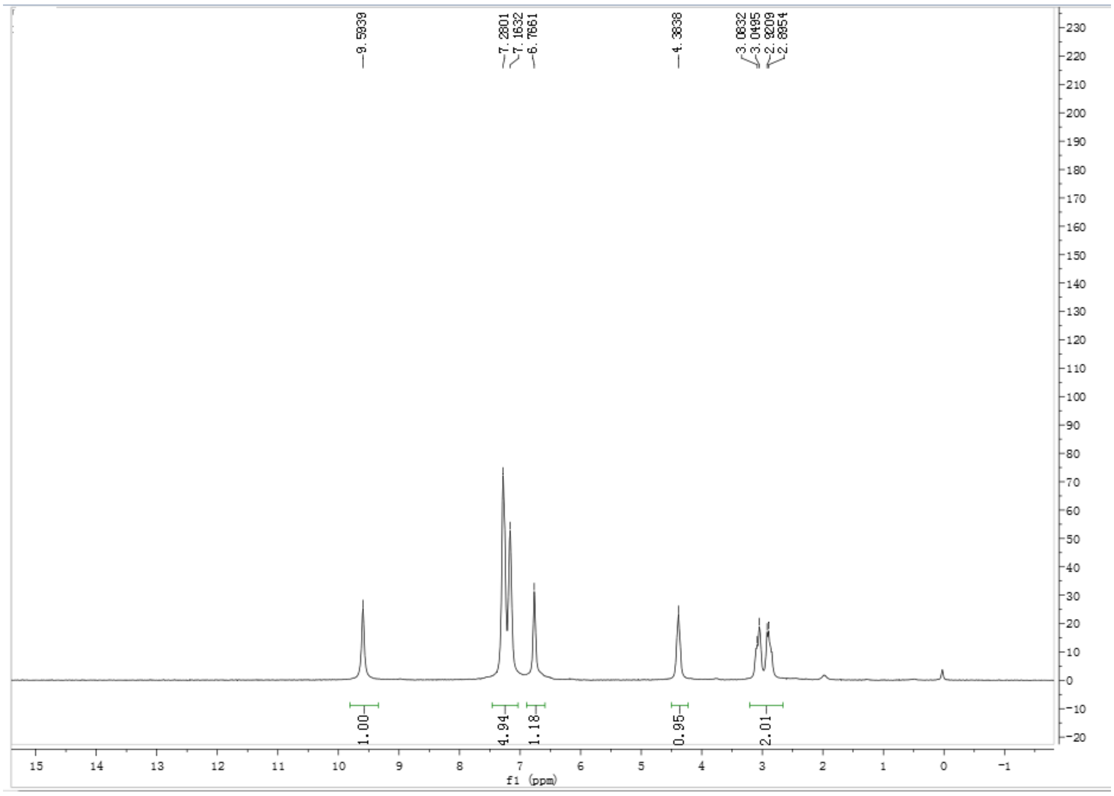

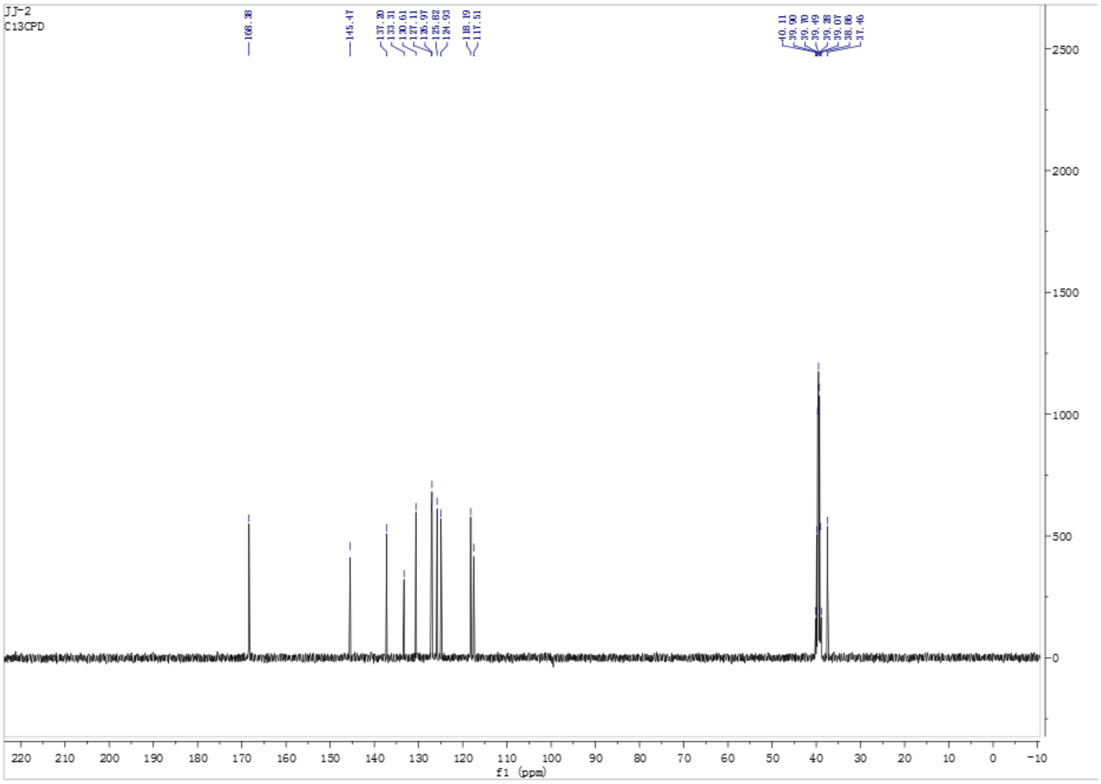

7-(4-Chlorophenyl)-6,7-dihydrothieno[3,2-*b*]pyridin-5(4*H*)-one (**1c**)

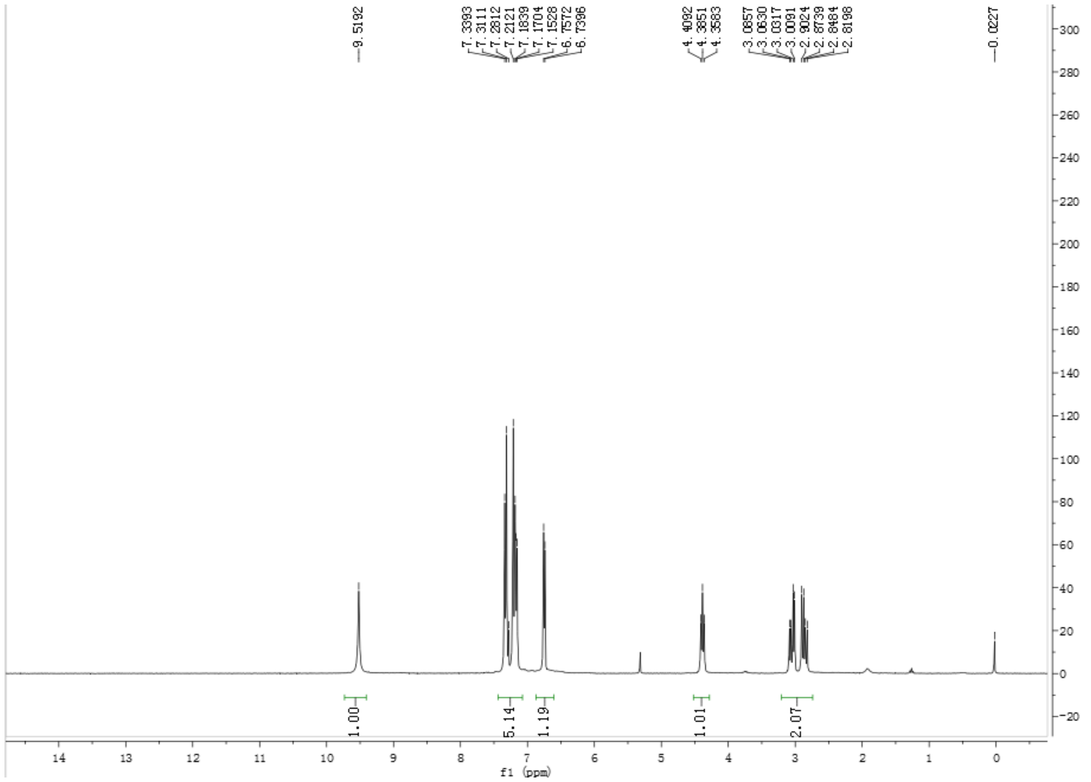

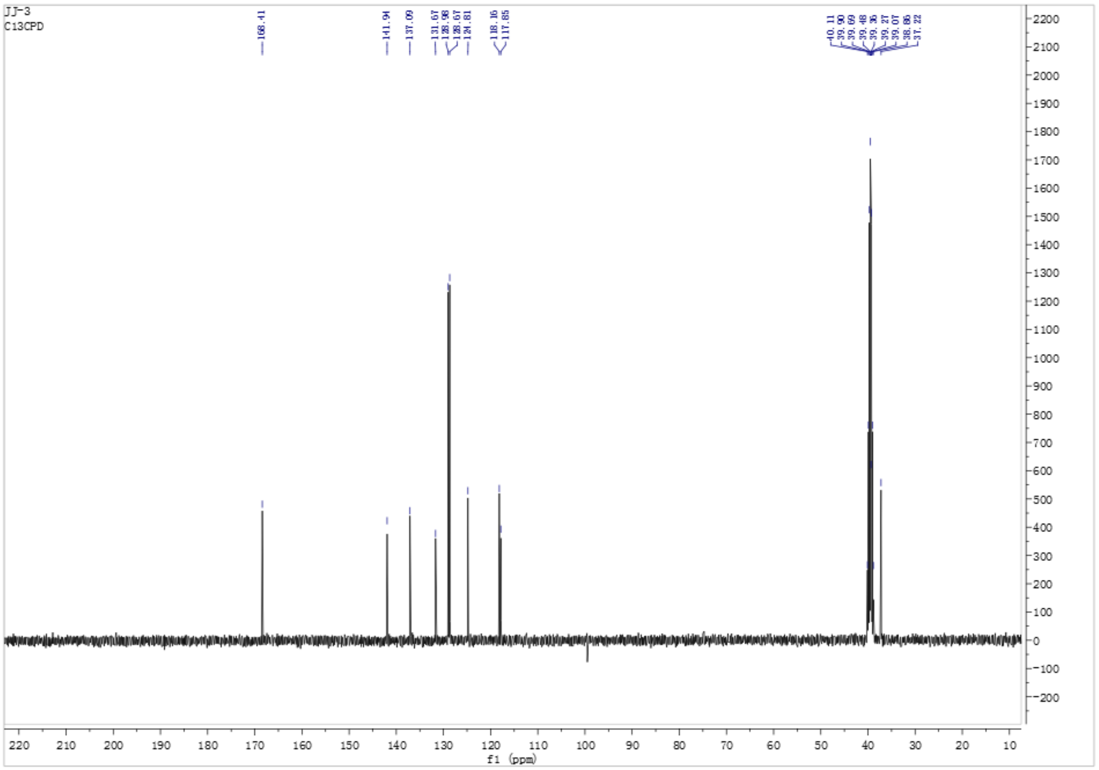

7-(2-Fluorophenyl)-6,7-dihydrothieno[3,2-*b*]pyridin-5(4*H*)-one (**1d**)

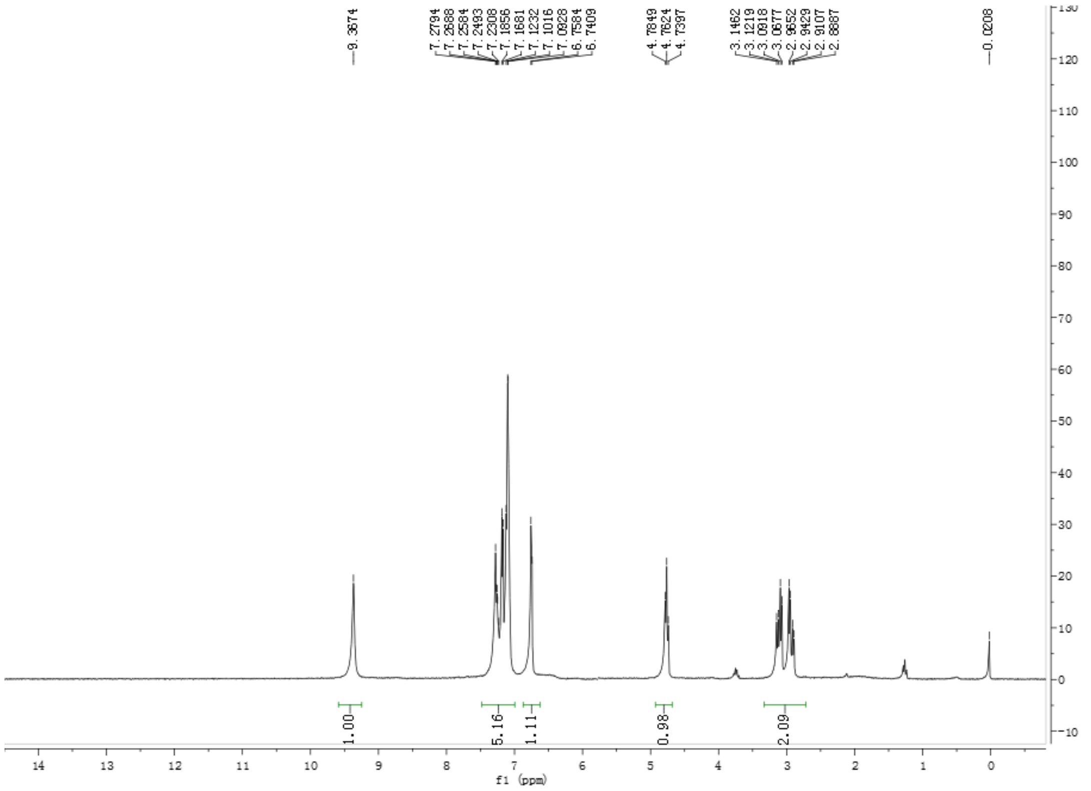

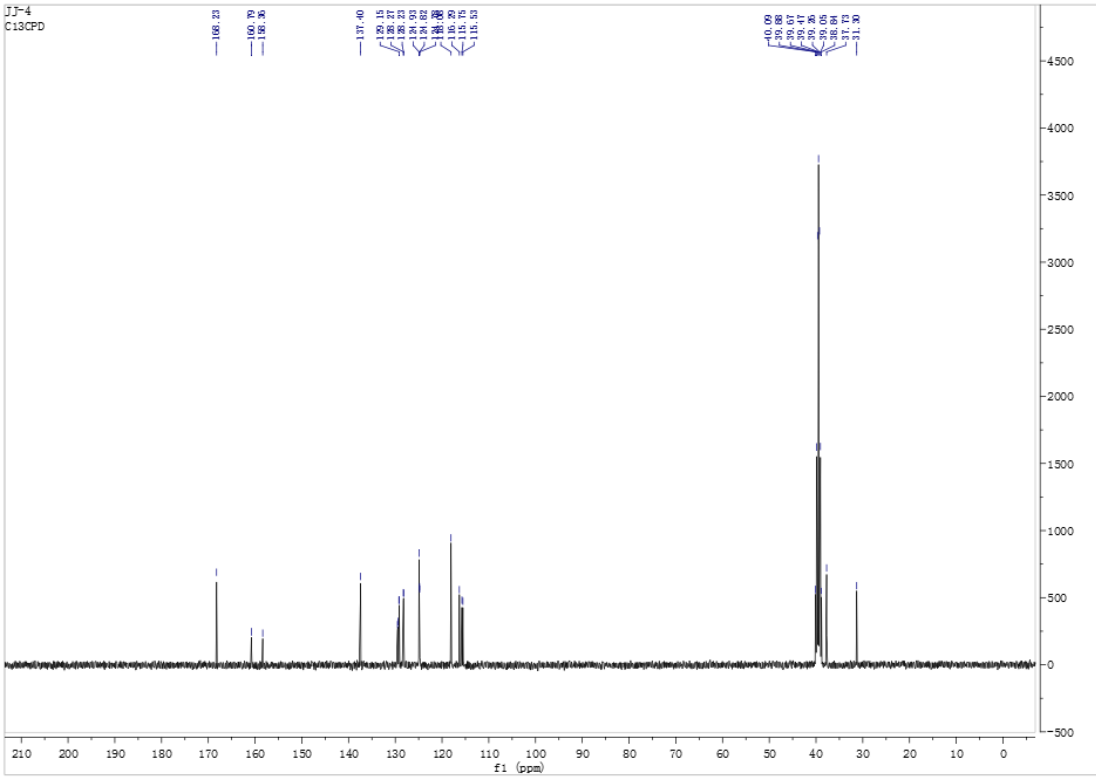

7-(3-Fluorophenyl)-6,7-dihydrothieno[3,2-*b*]pyridin-5(4*H*)-one (**1e**)

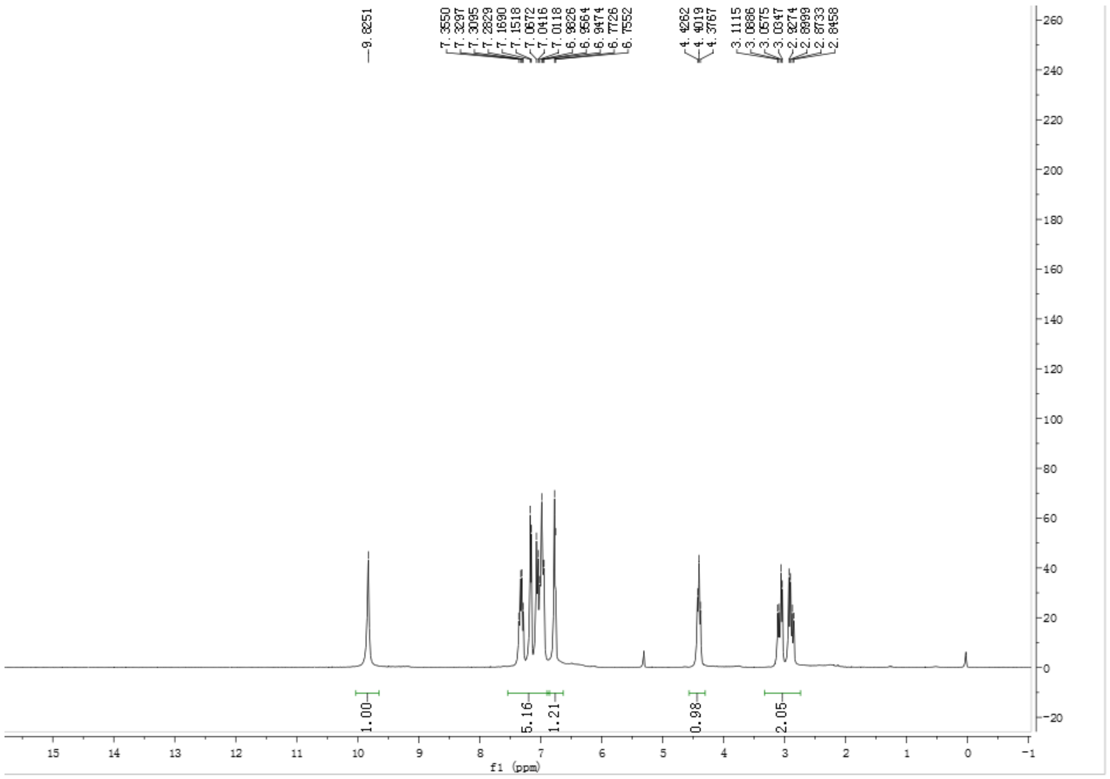

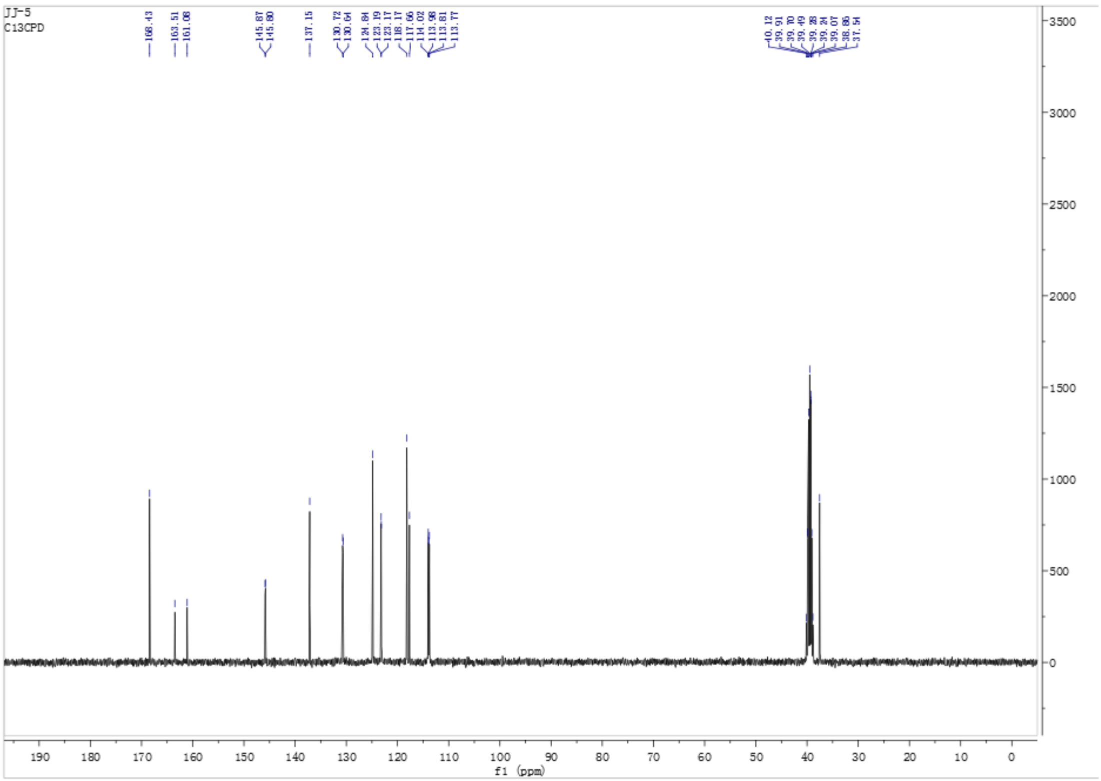

7-(4-Fluorophenyl)-6,7-dihydrothieno[3,2-*b*]pyridin-5(4*H*)-one (**1f**)

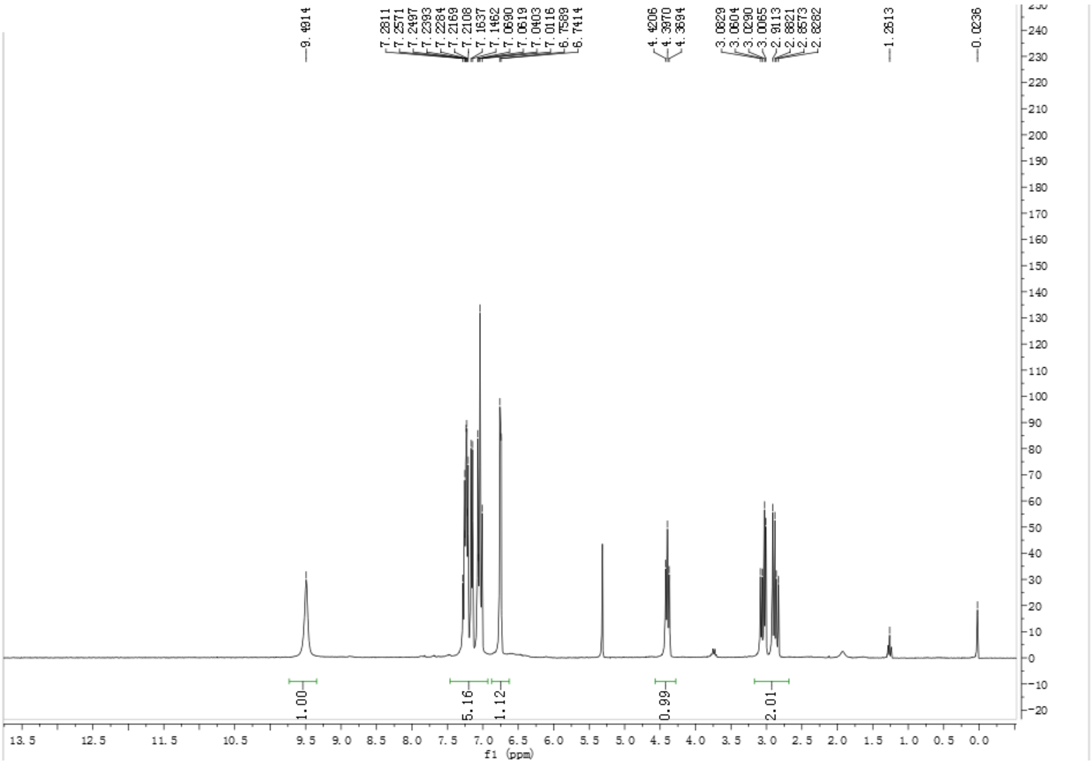

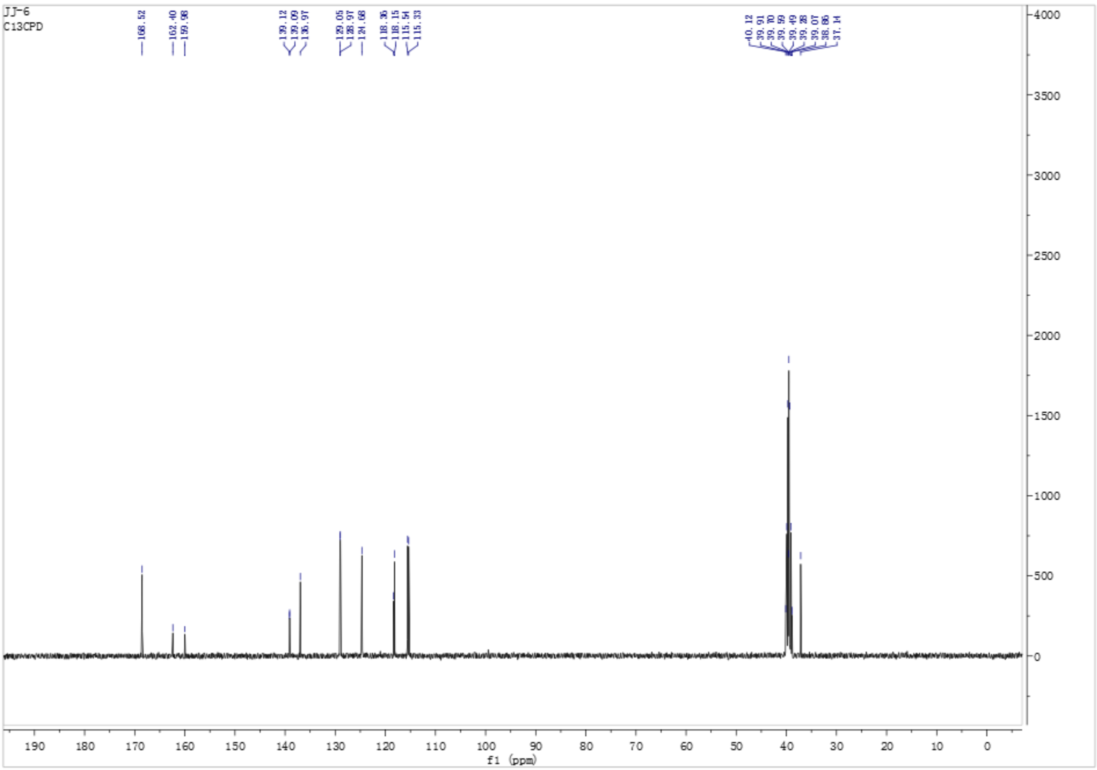

7-[2-(Trifluoromethyl)phenyl]-6,7-dihydrothieno[3,2-*b*]pyridin-5(4*H*)-one (**1g**)

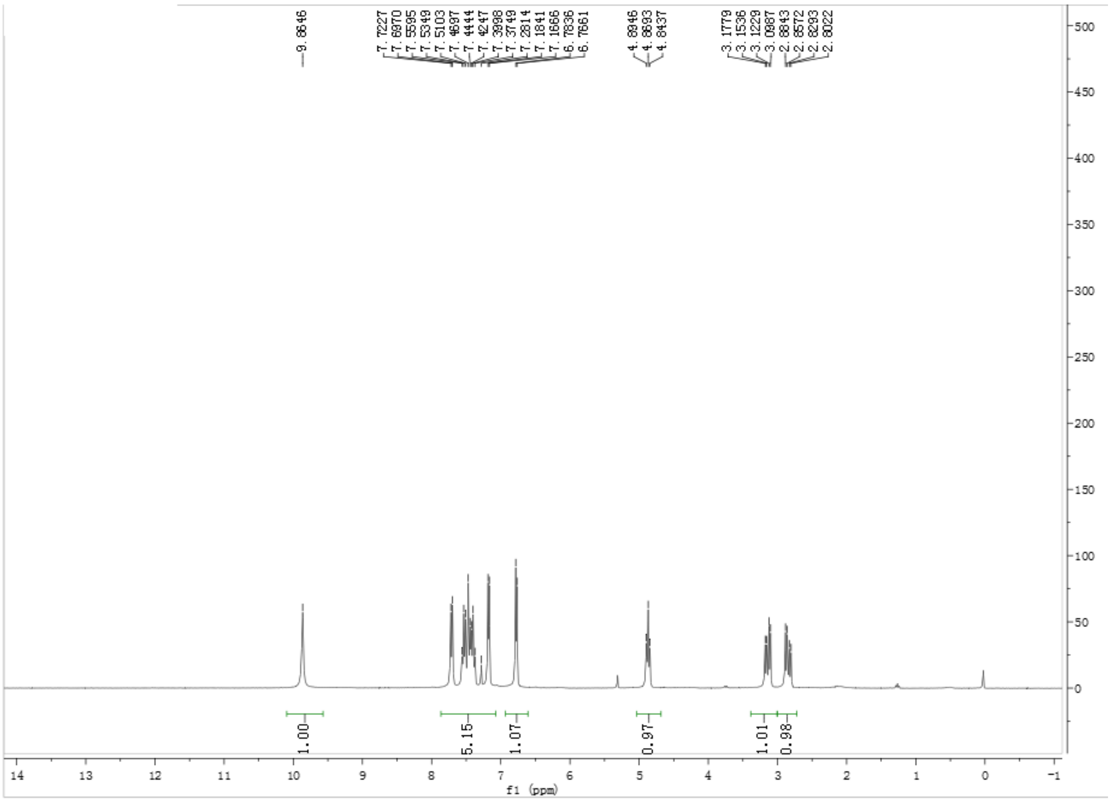

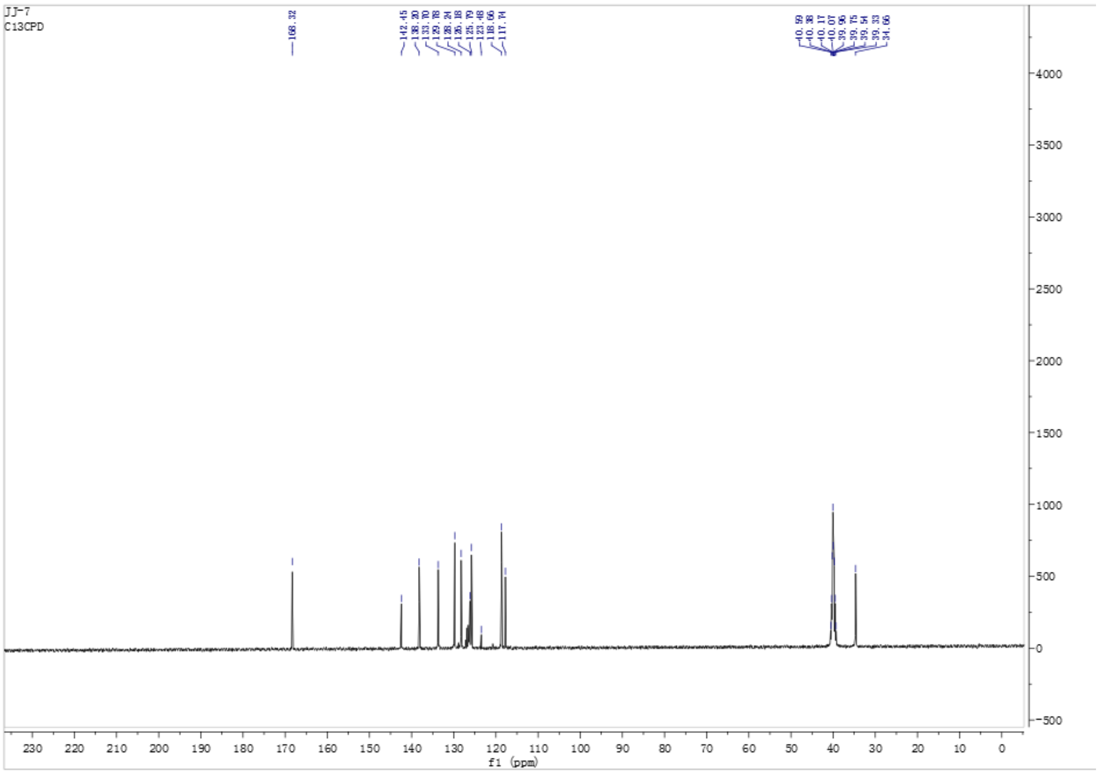

7-[3-(Trifluoromethyl)phenyl]-6,7-dihydrothieno[3,2-*b*]pyridin-5(4*H*)-one (**1h**)

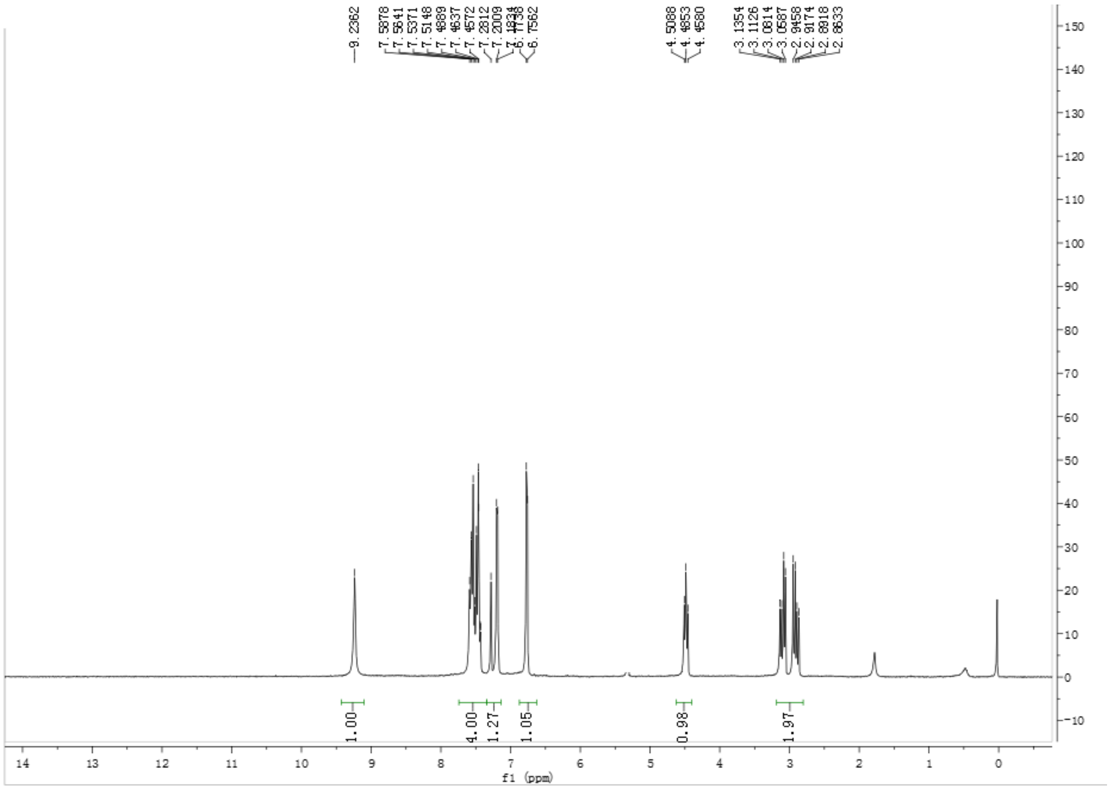

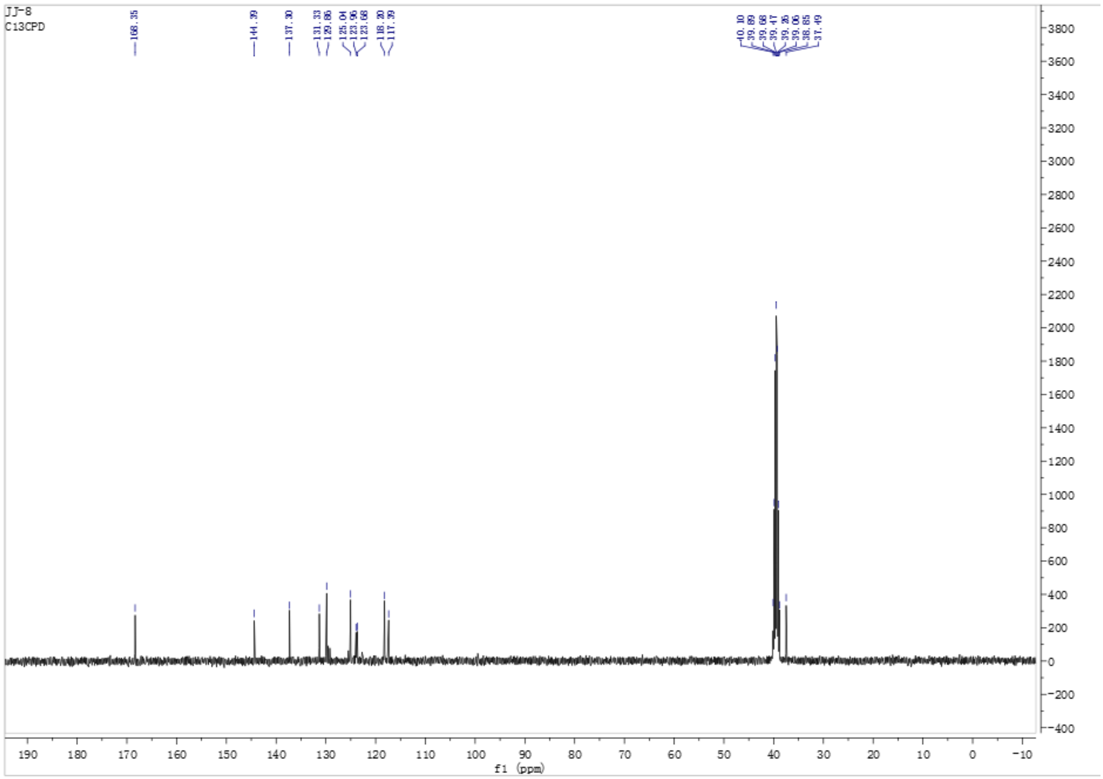

7-[4-(Trifluoromethyl)phenyl]-6,7-dihydrothieno[3,2-*b*]pyridin-5(4*H*)-one (**1i**)

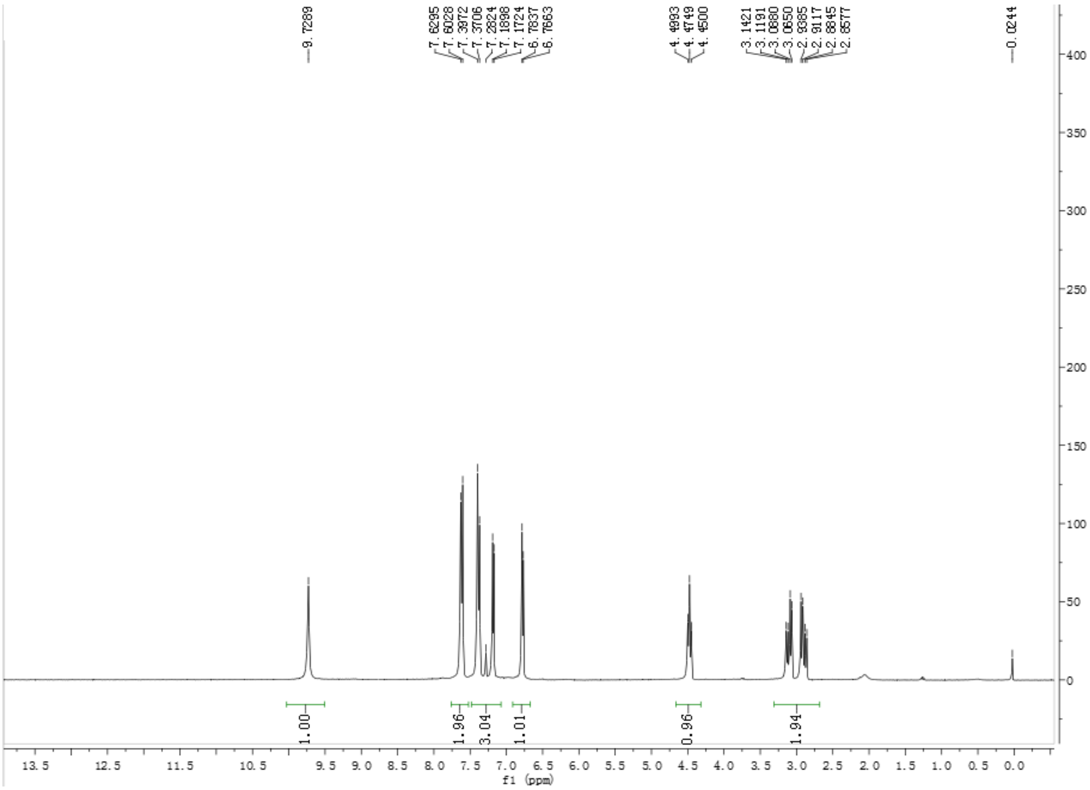

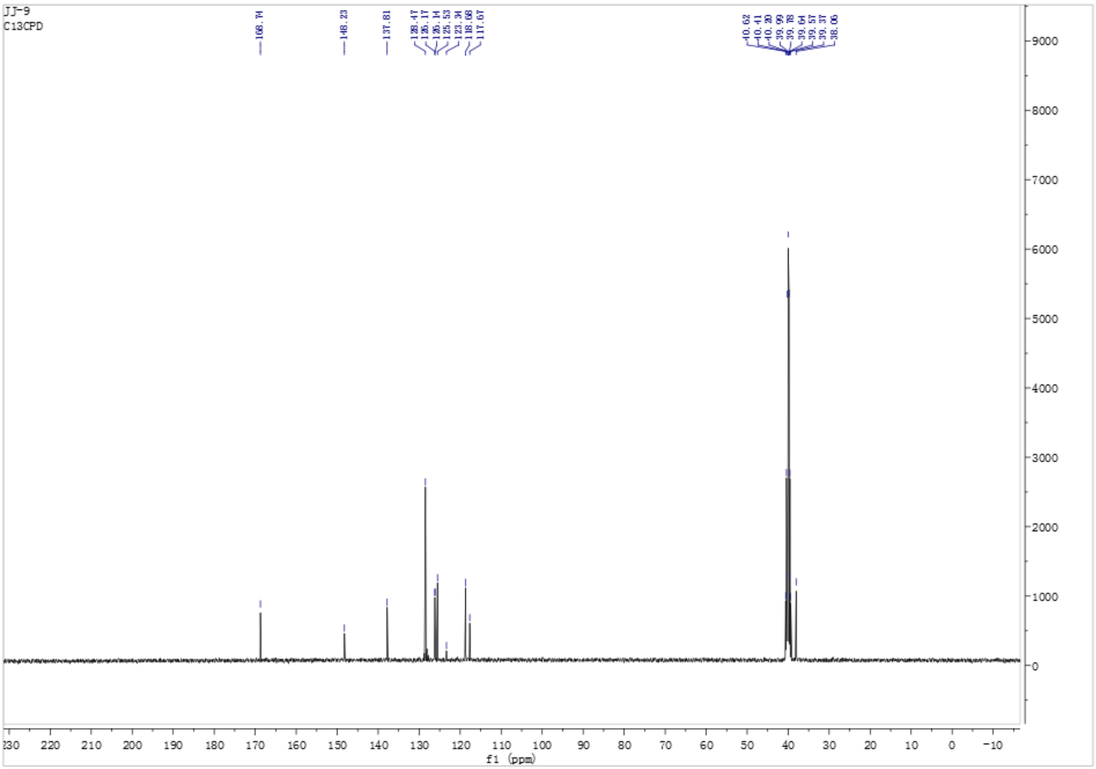

7-(2-Methoxyphenyl)-6,7-dihydrothieno[3,2-*b*]pyridin-5(4*H*)-one (**1j**)

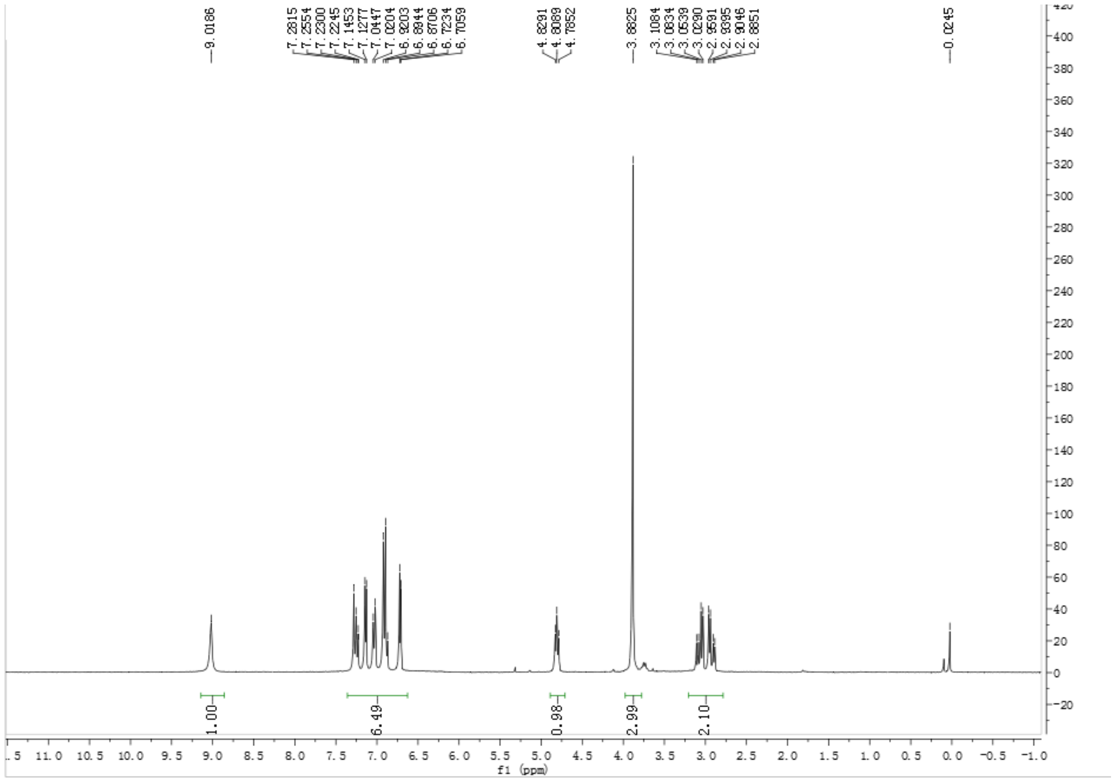

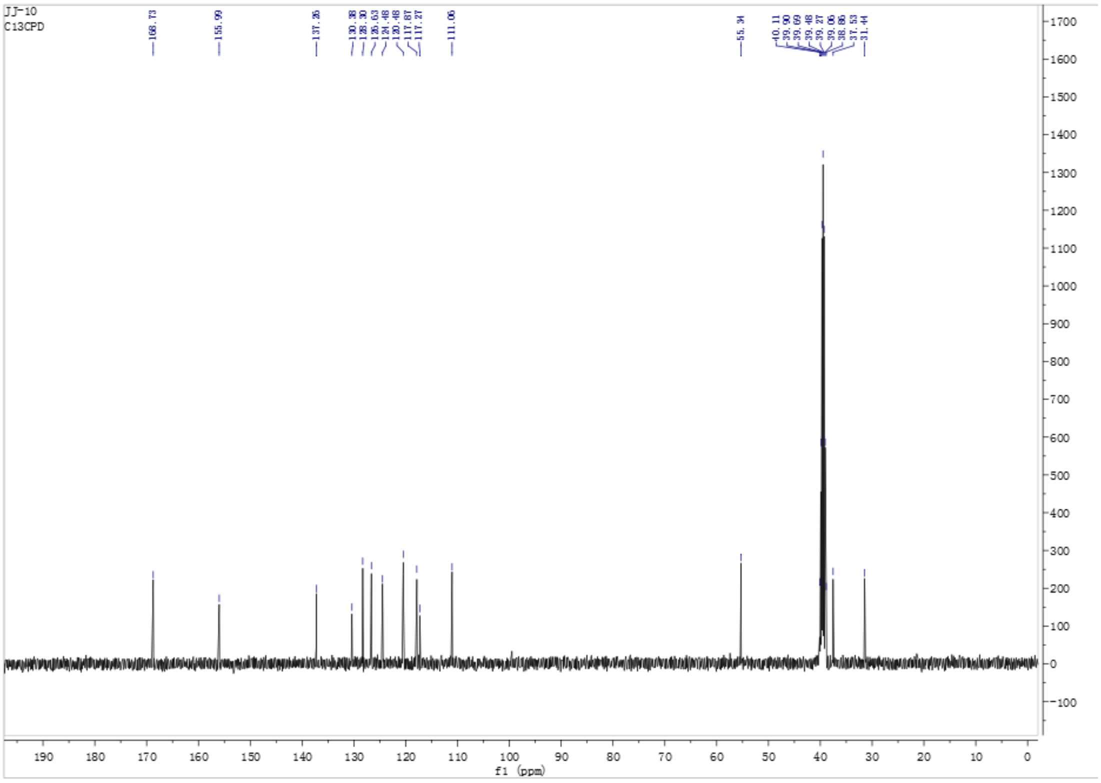

7-(3-Methoxyphenyl)-6,7-dihydrothieno[3,2-*b*]pyridin-5(4*H*)-one (**1k**)

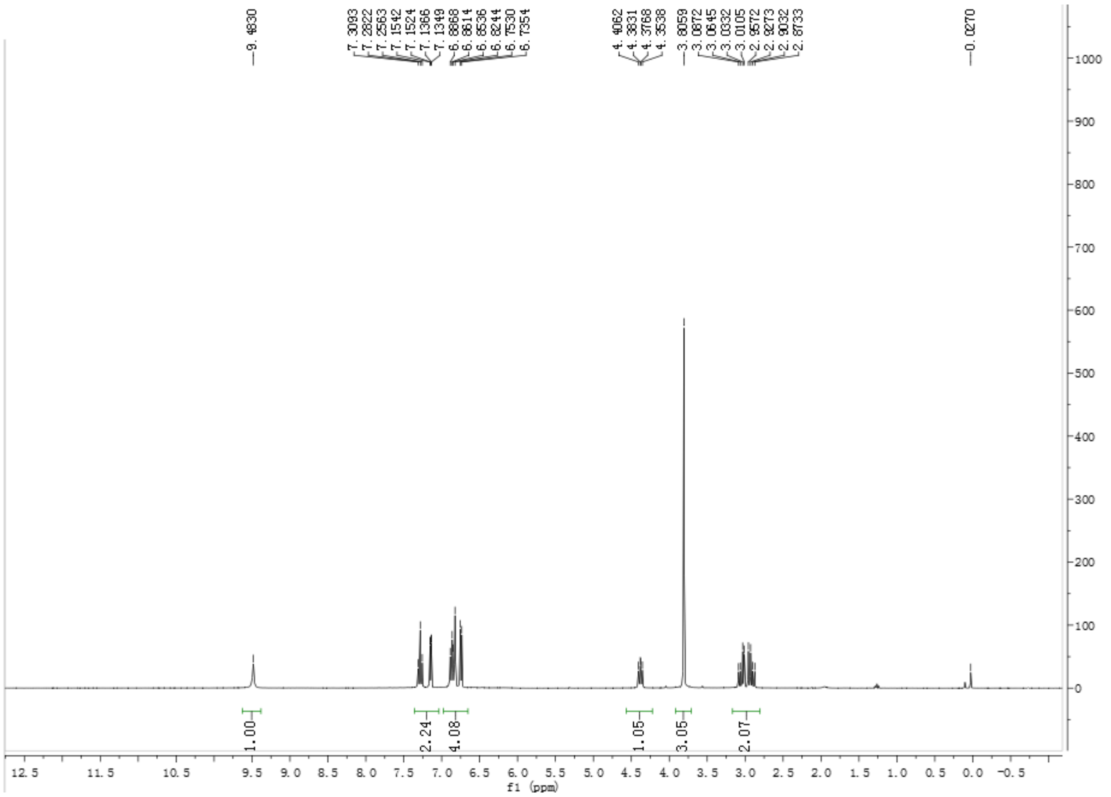

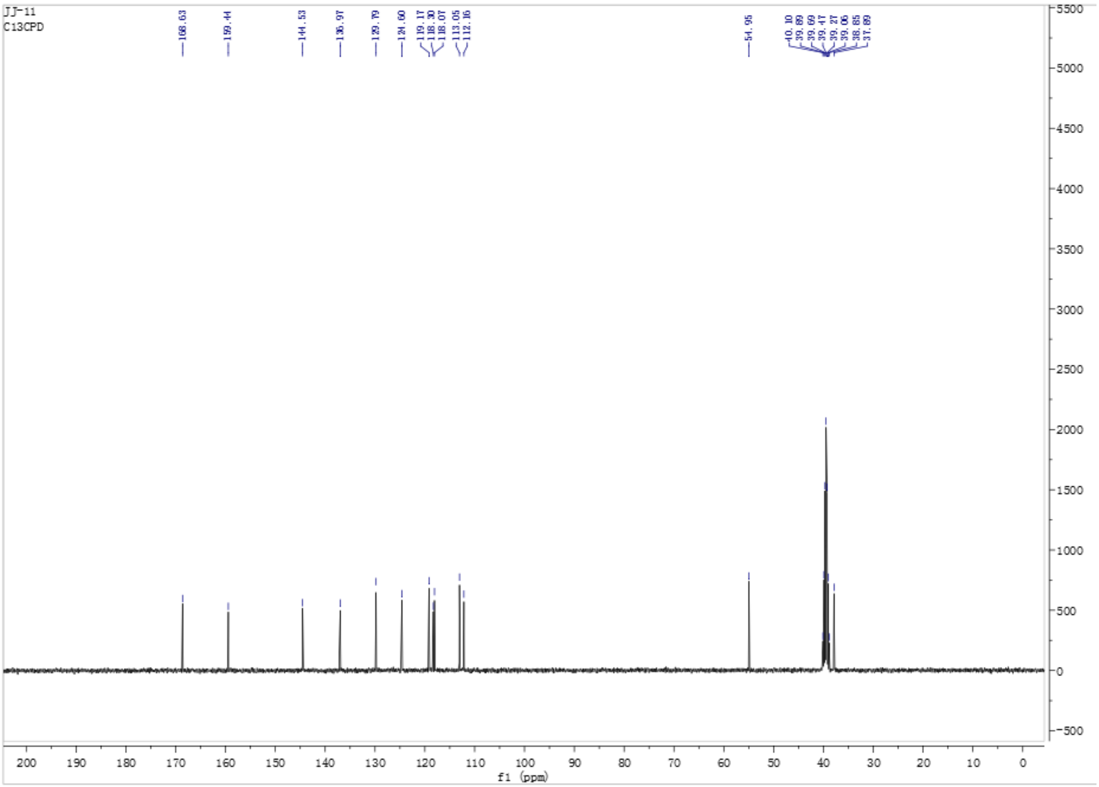

7-(4-Methoxyphenyl)-6,7-dihydrothieno[3,2-*b*]pyridin-5(4*H*)-one (**1l**)

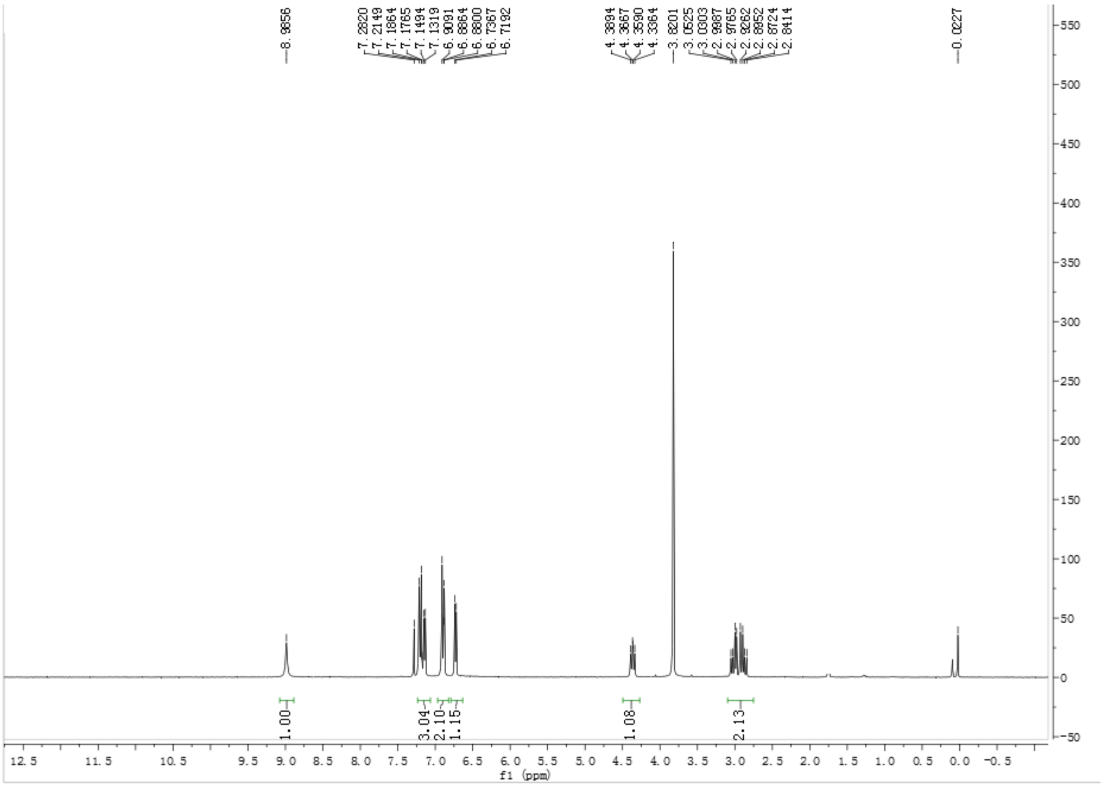

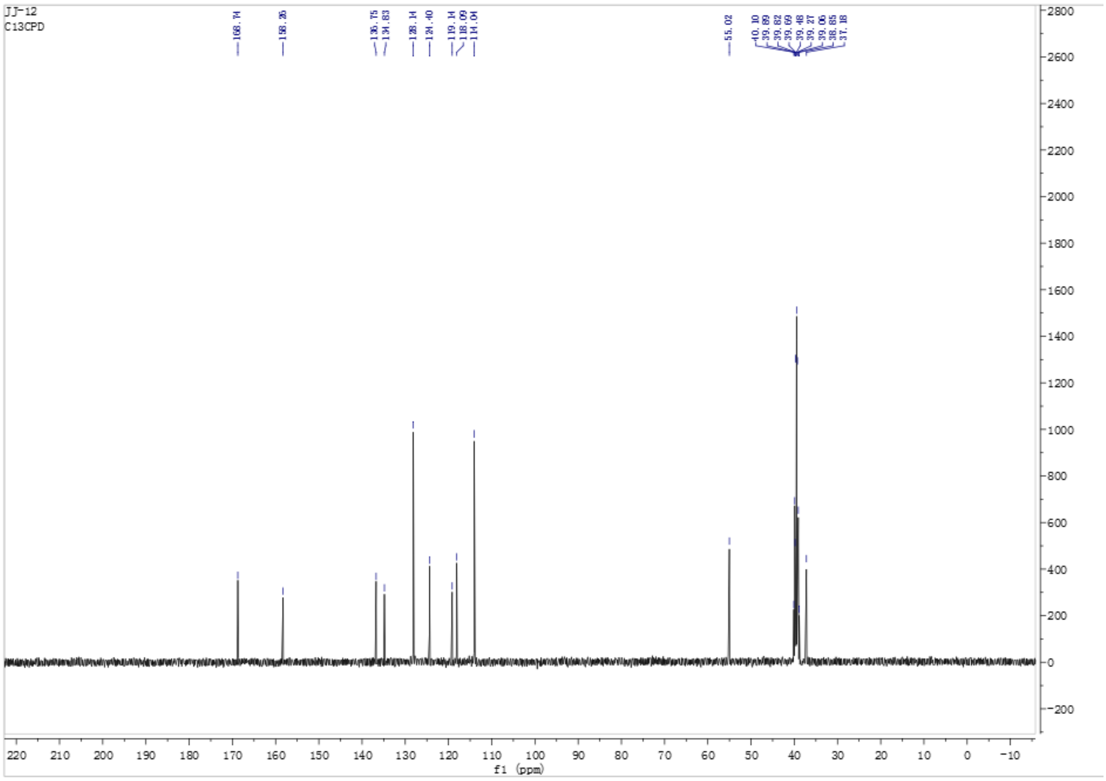

7-(4-Bromophenyl)-6,7-dihydrothieno[3,2-*b*]pyridin-5(4*H*)-one (**1m**)

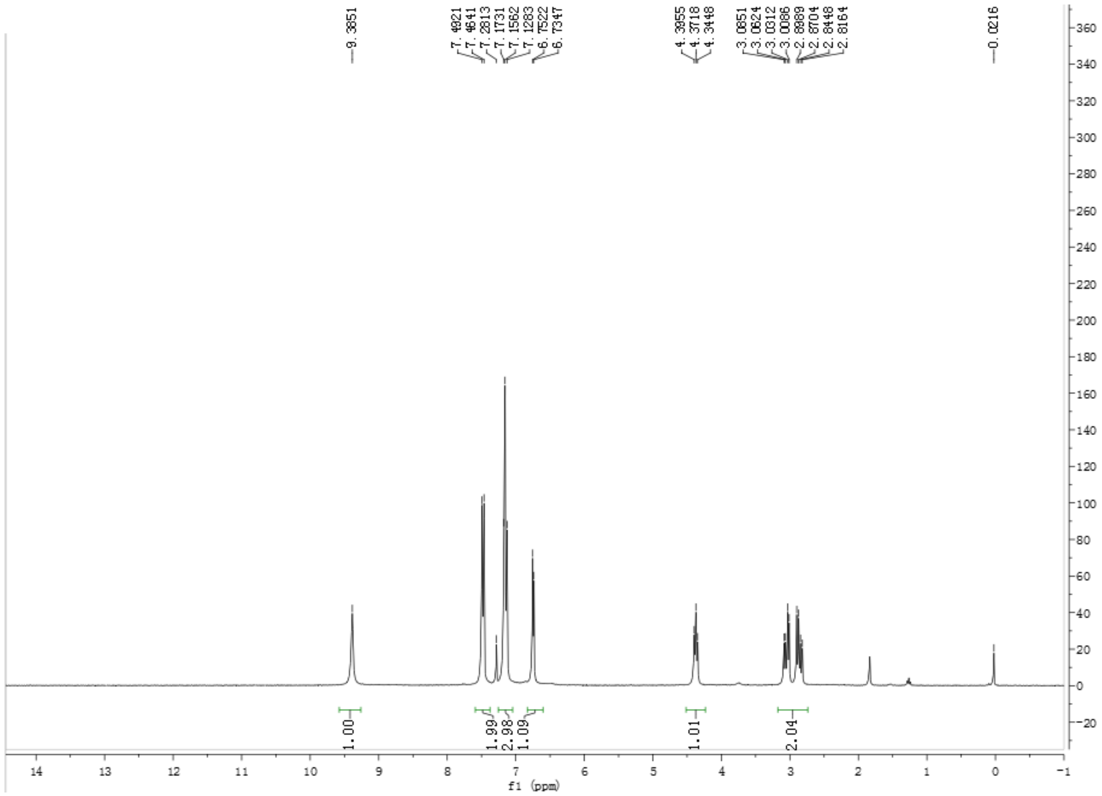

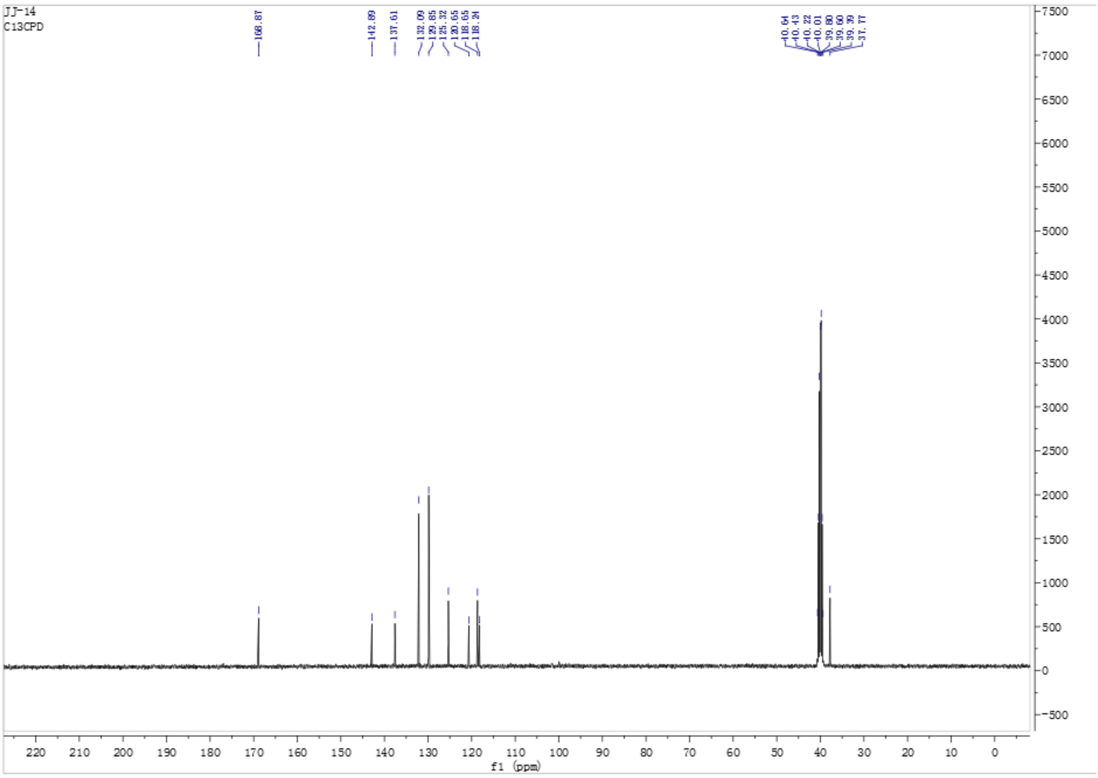

7-Phenyl-6,7-dihydrothieno[3,2-*b*]pyridin-5(4*H*)-one (**1n**)

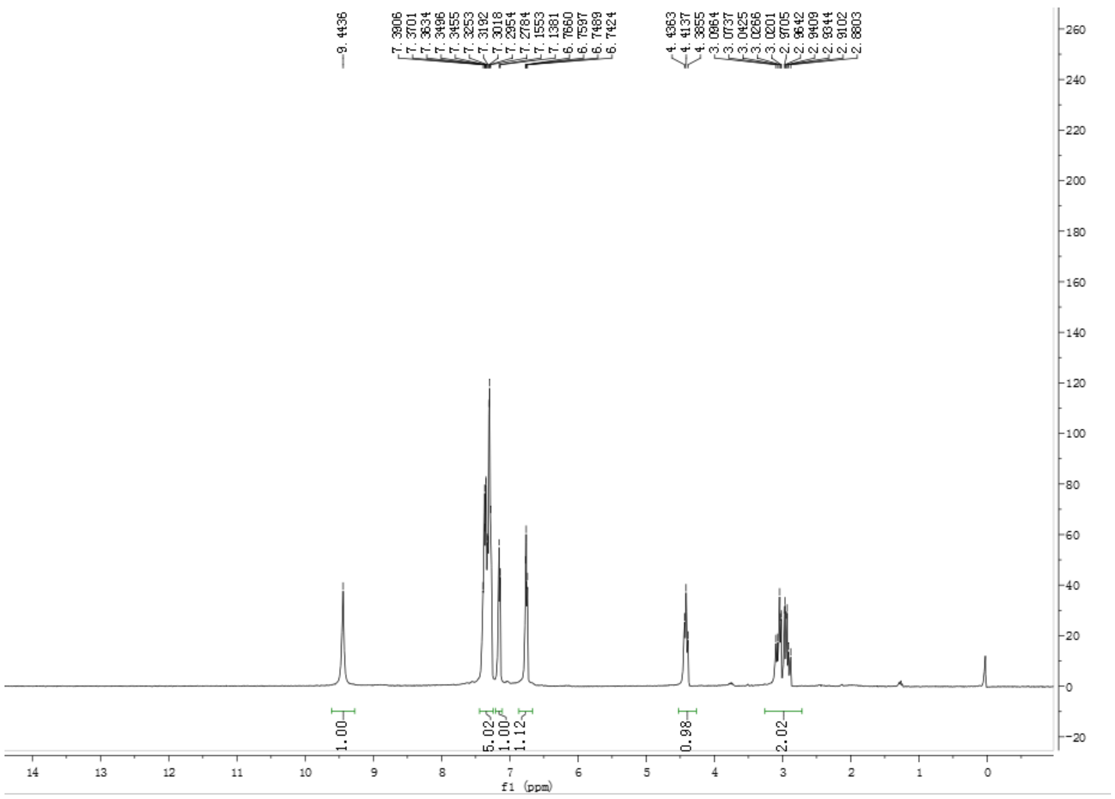

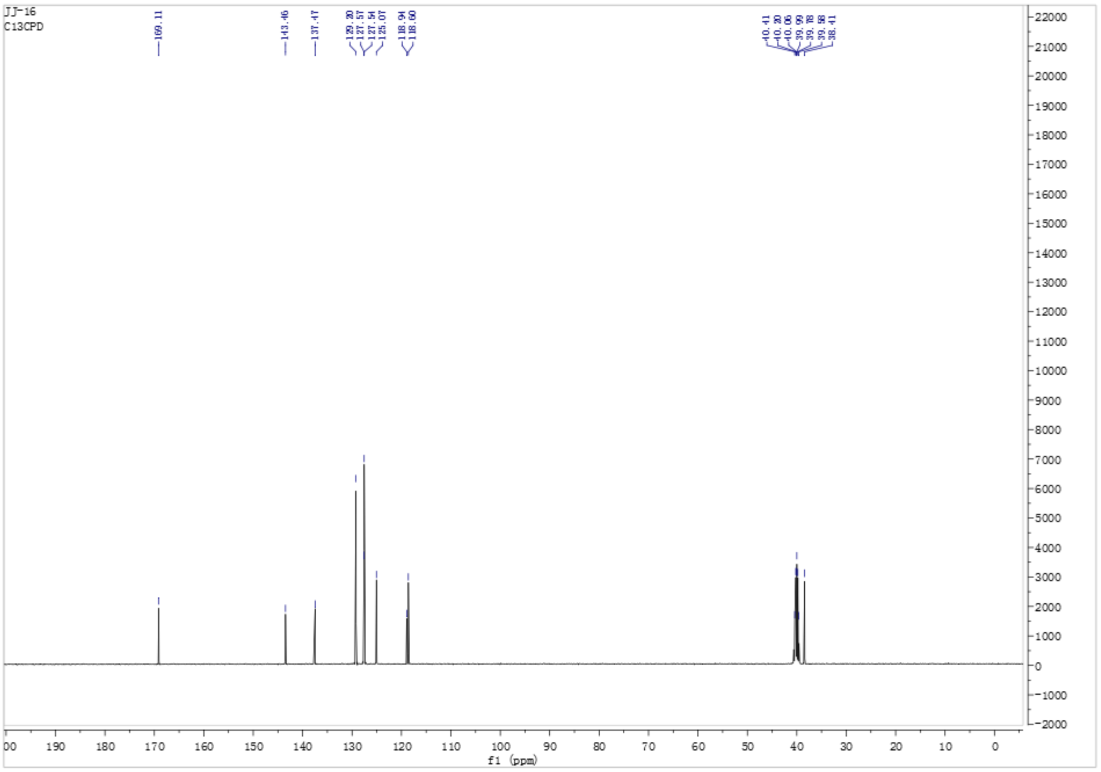

7-(2-Chloro-6-fluorophenyl)-6,7-dihydrothieno[3,2-*b*]pyridin-5(4*H*)-one (**1o**)

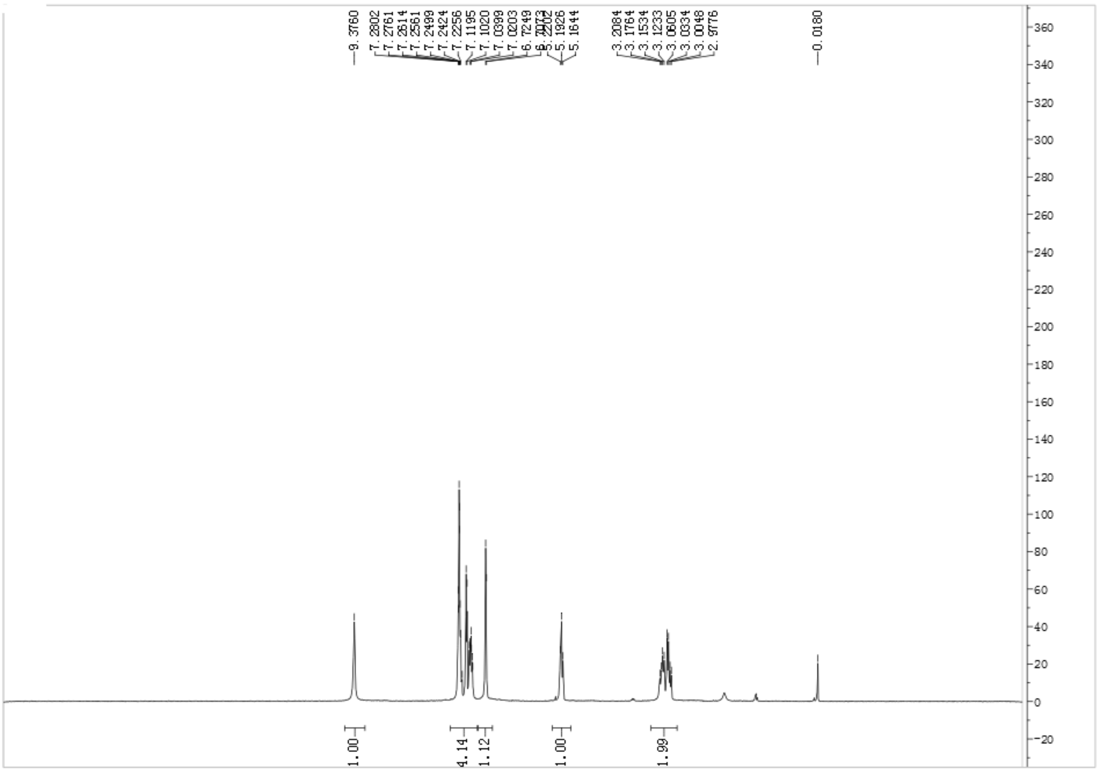

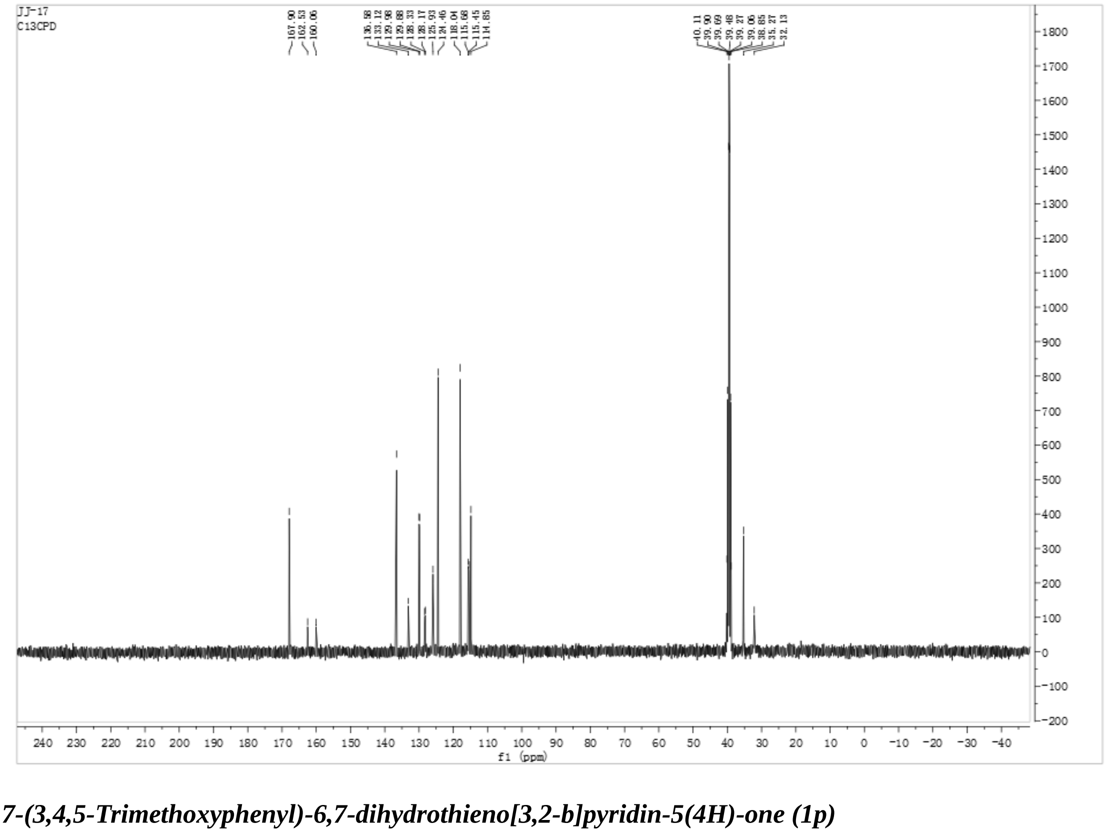

7-(3,4,5-Trimethoxyphenyl)-6,7-dihydrothieno[3,2-b]pyridin-5(4H)-one (1p)

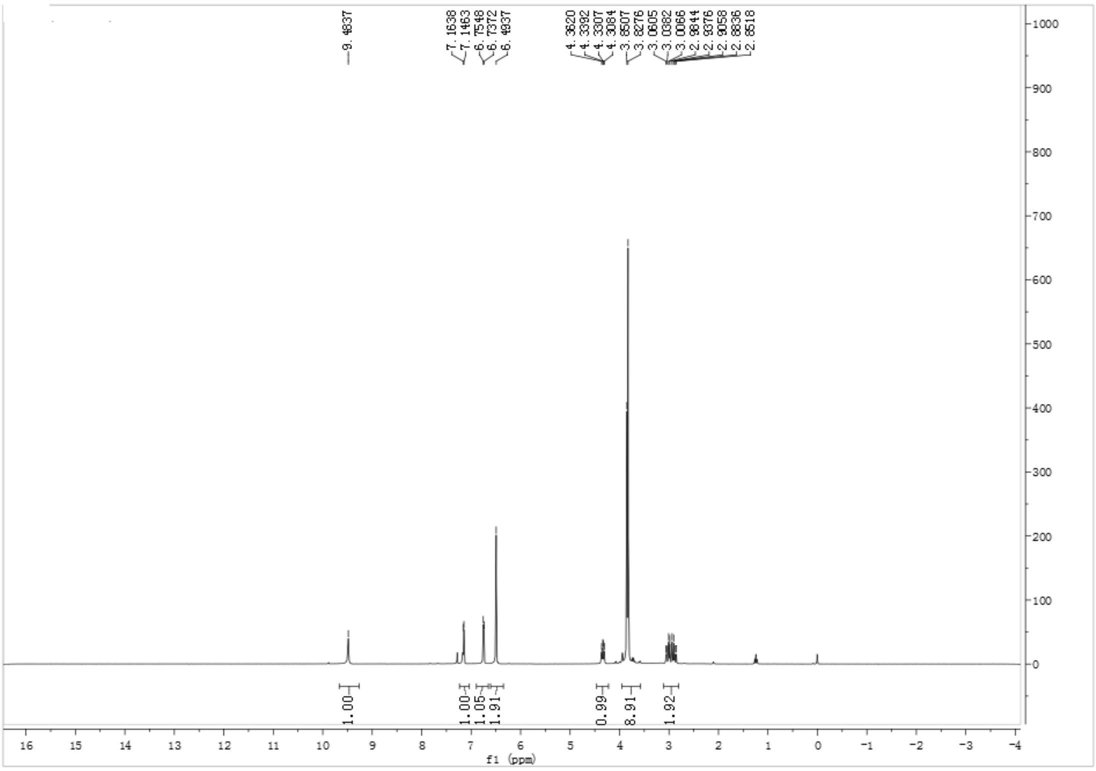

9.4837
7.1638
7.1463
6.7548
6.7372
6.4937
4.3620
4.3392
4.3307
4.3084
3.8507
3.8276
3.0605
3.0382
3.0066
2.9844
2.9376
2.9058
2.8836
2.8518
1.00
1.00
1.05
1.91
0.99
8.91
1.92
f1 (ppm)

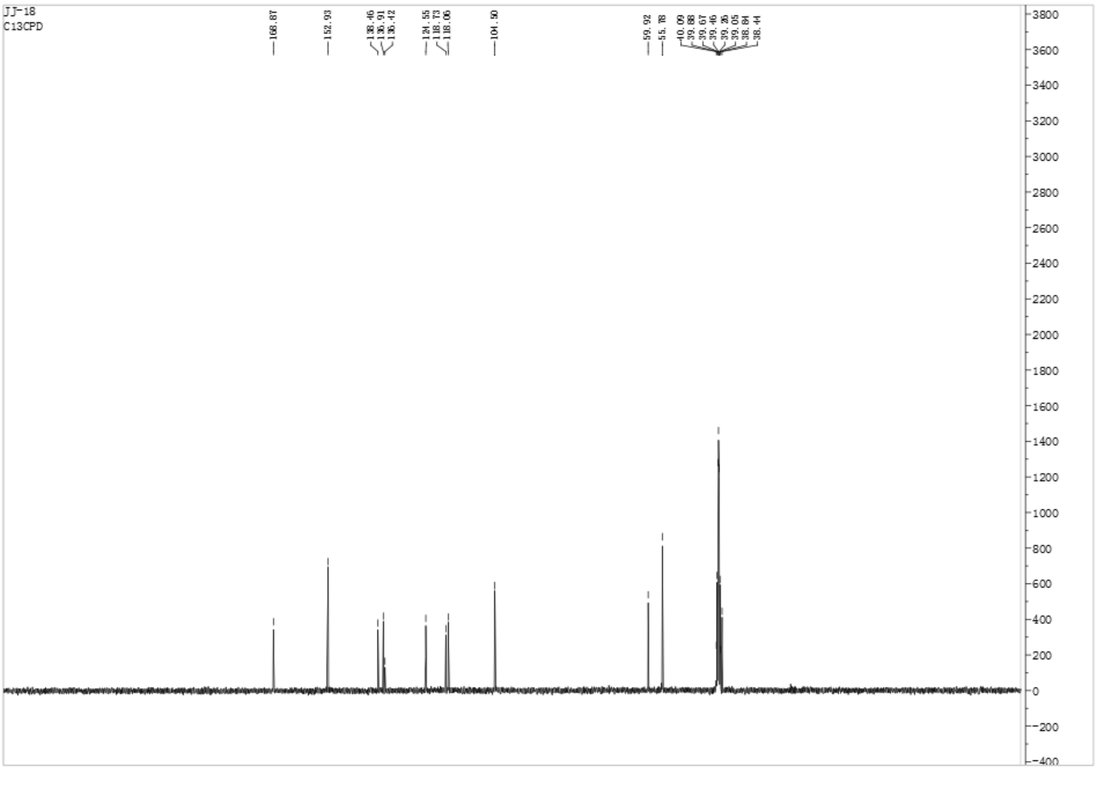

JJ-18
C13CPD
166.87
152.93
138.46
136.91
136.42
124.55
118.73
118.06
104.90
59.92
55.78
40.09
39.88
39.67
39.46
39.26
39.05
38.84
38.44

Target compounds 4a-p ^{1}H-NMR and ^{13}C-NMR spectrum:

5-(2-Chlorophenyl)-4,5-dihydrotetrazolo[1,5-*a*]thieno[2,3-*e*]pyridine **(4a)**

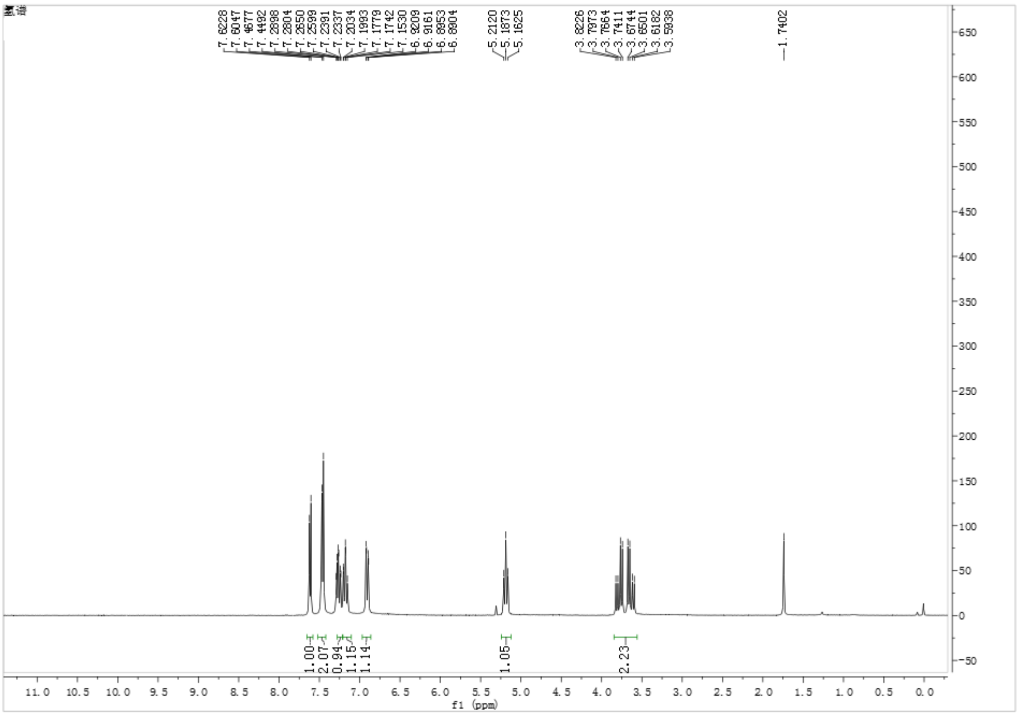

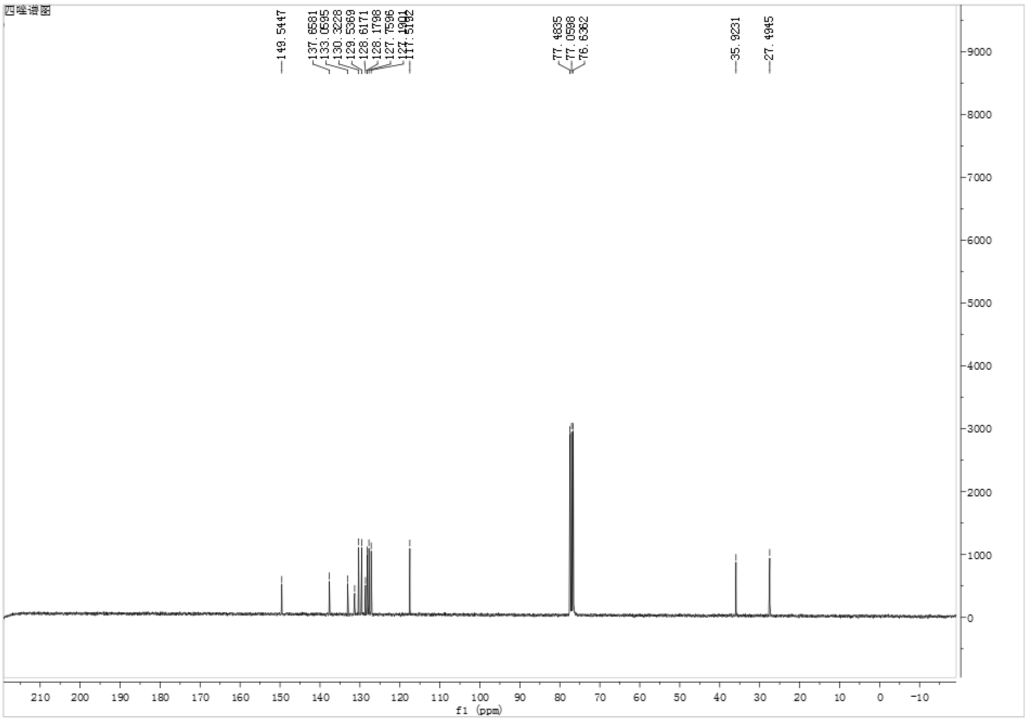

5-(3-Chlorophenyl)-4,5-dihydrotetrazolo[1,5-a]thieno[2,3-e]pyridine(**4b**)

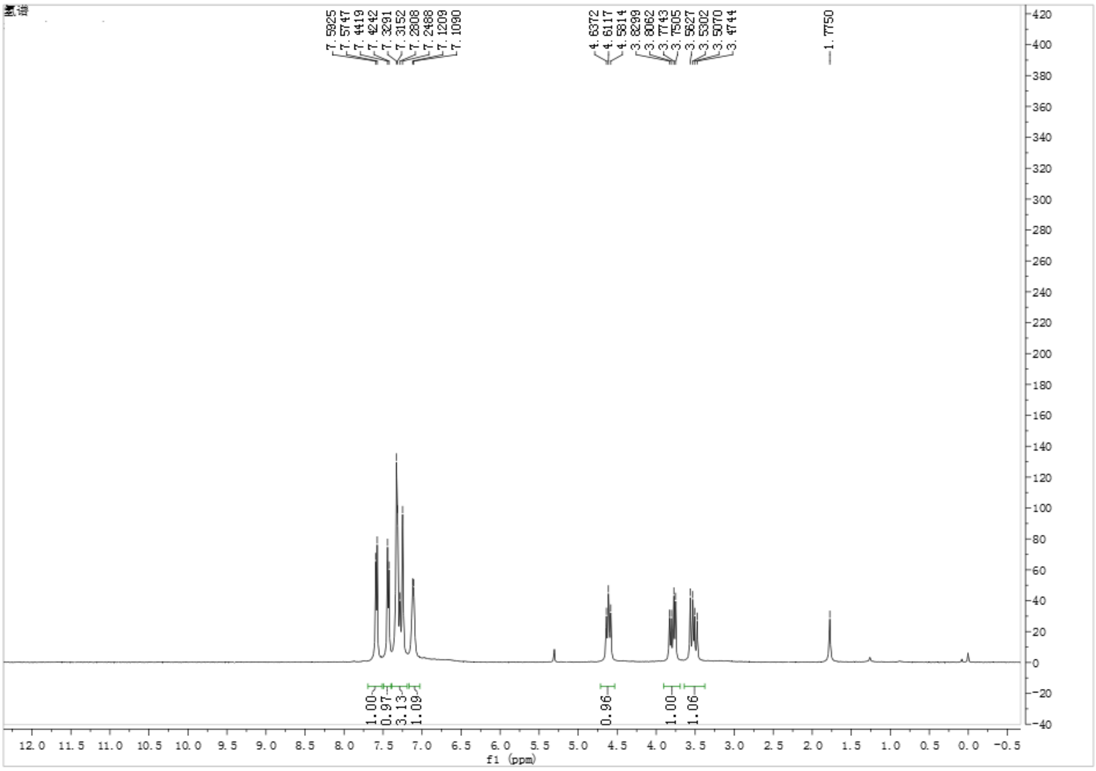

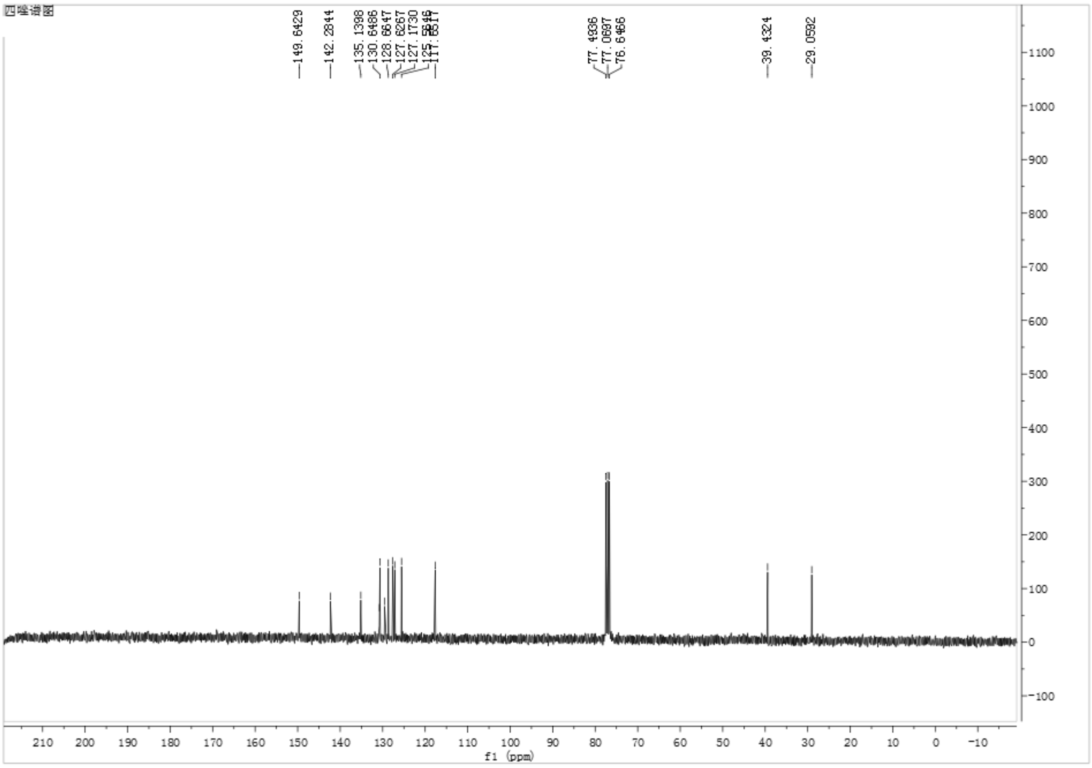

5-(4-Chlorophenyl)-4,5-dihydrotetrazolo[1,5-a]thieno[2,3-e]pyridine (**4c**)

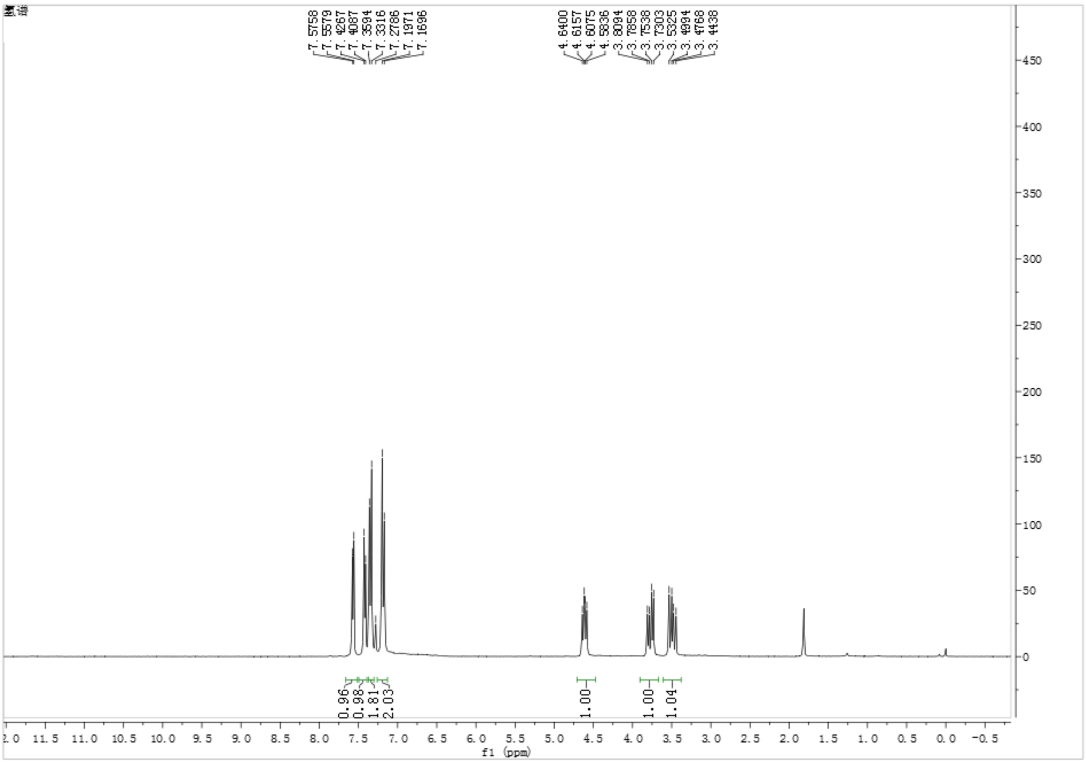

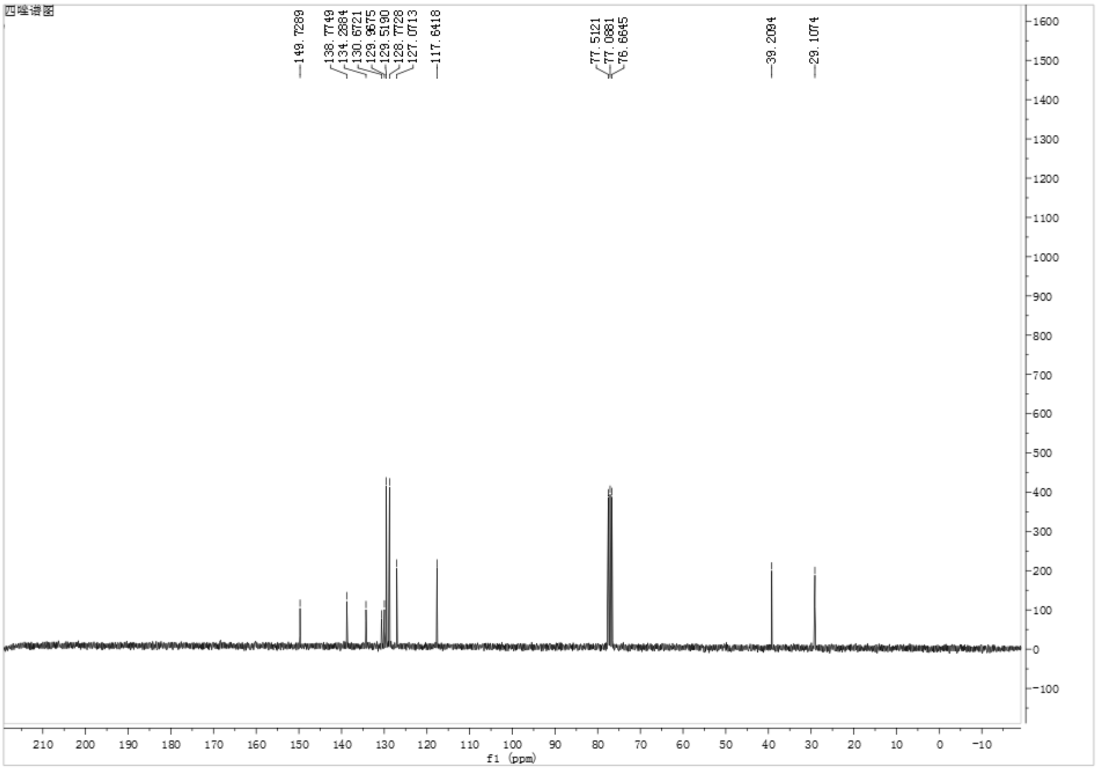

5-(2-Flulorophenyl)-4,5-dihydrotetrazolo[1,5-a]thieno[2,3-e]pyridine (**4d**)

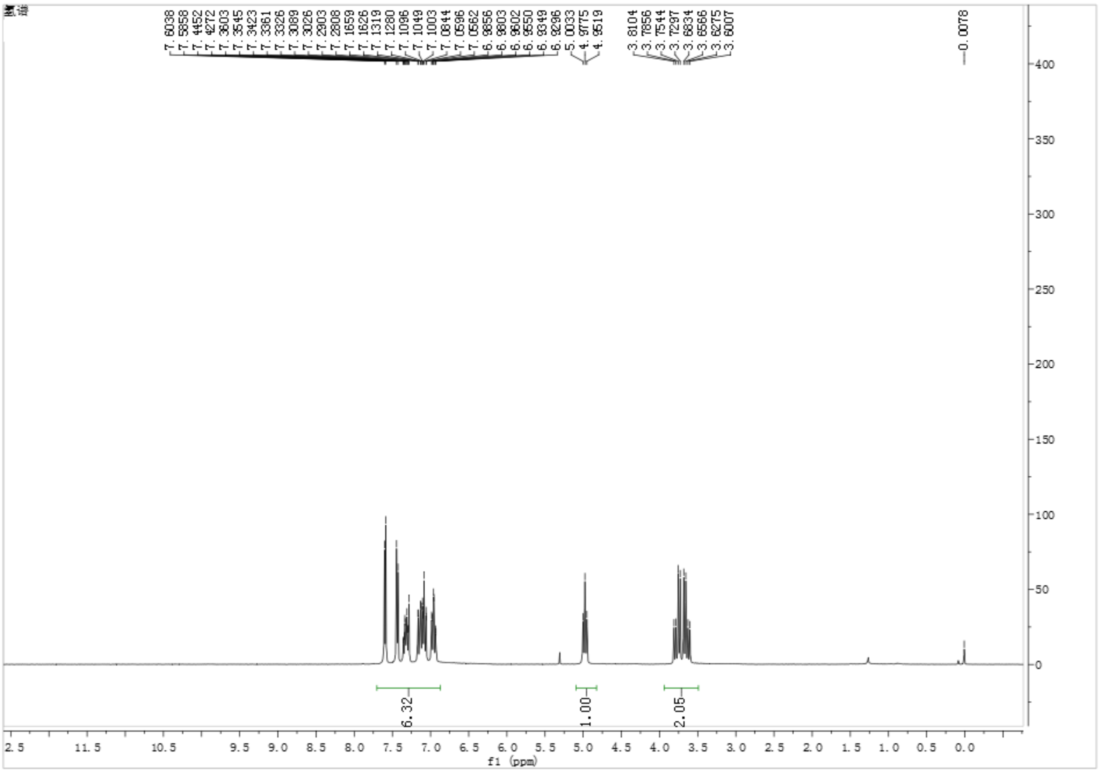

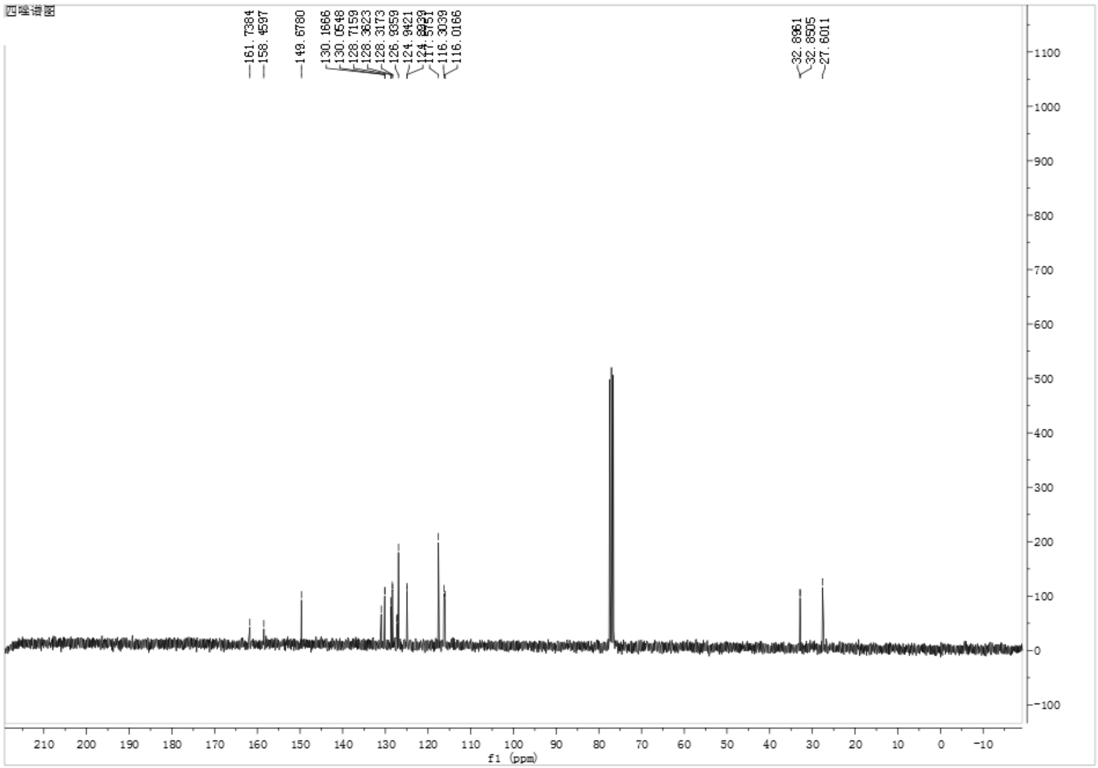

5-(3-Flulorophenyl)-4,5-dihydrotetrazolo[1,5-a]thieno[2,3-e]pyridine (**4e**)

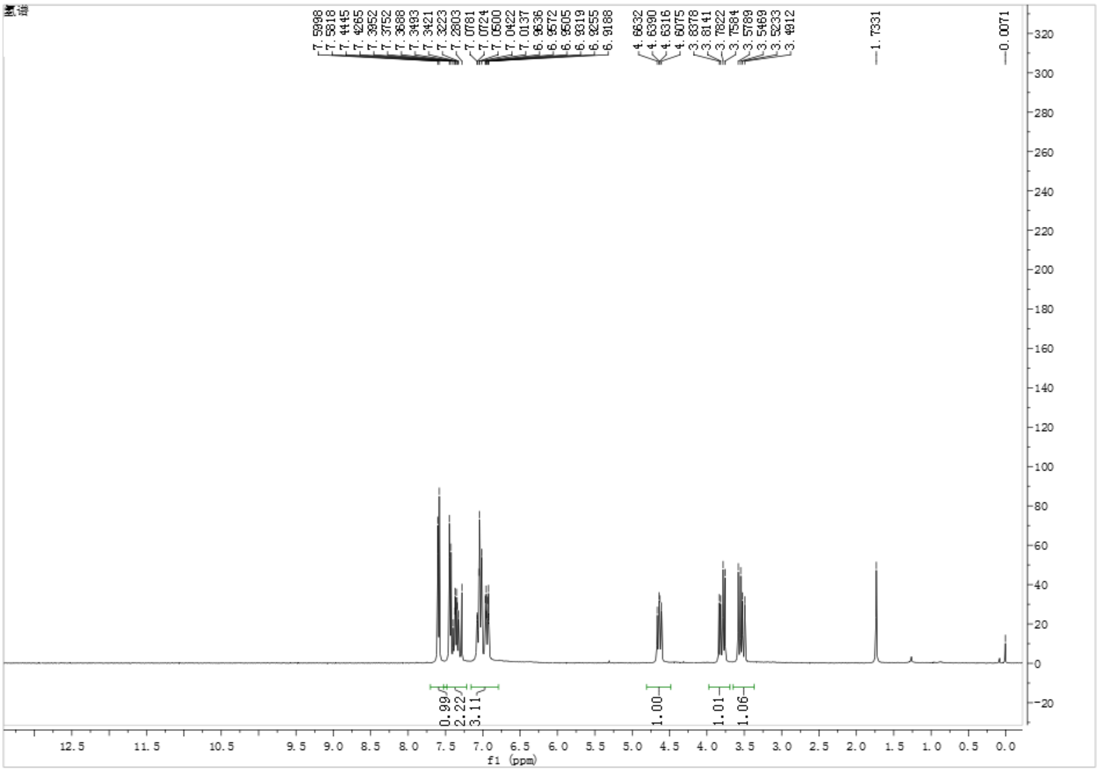

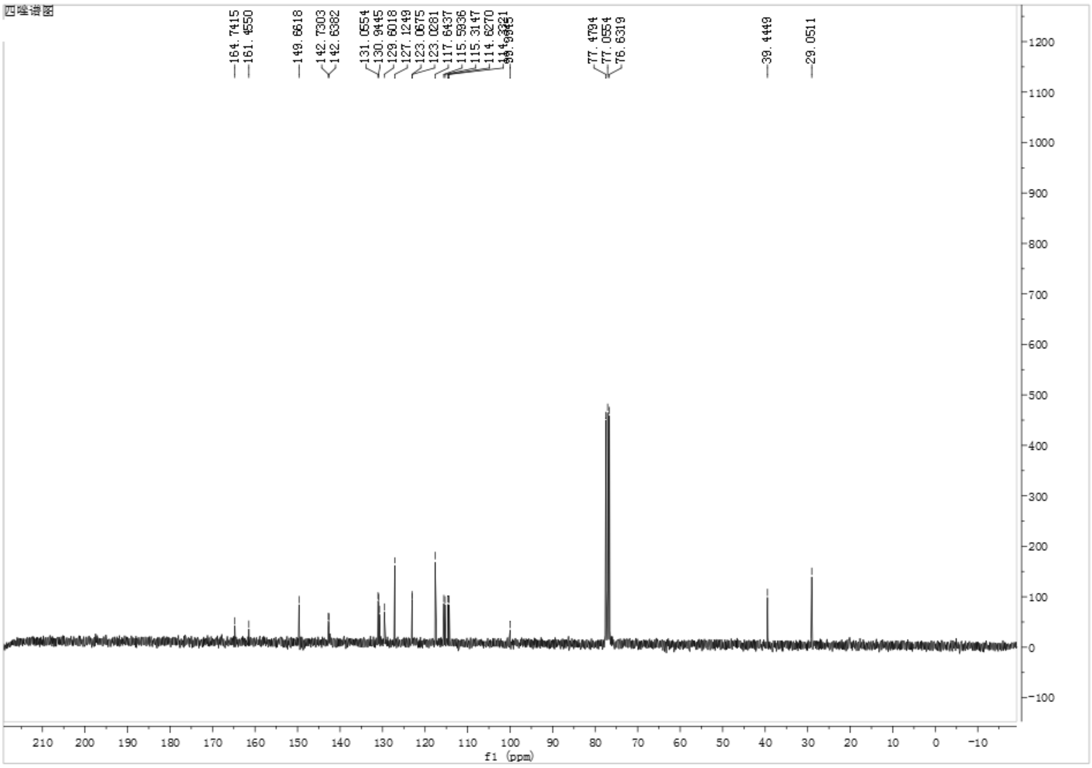

5-(4-Flulorophenyl)-4,5-dihydrotetrazolo[1,5-a]thieno[2,3-e]pyridine (**4f**)

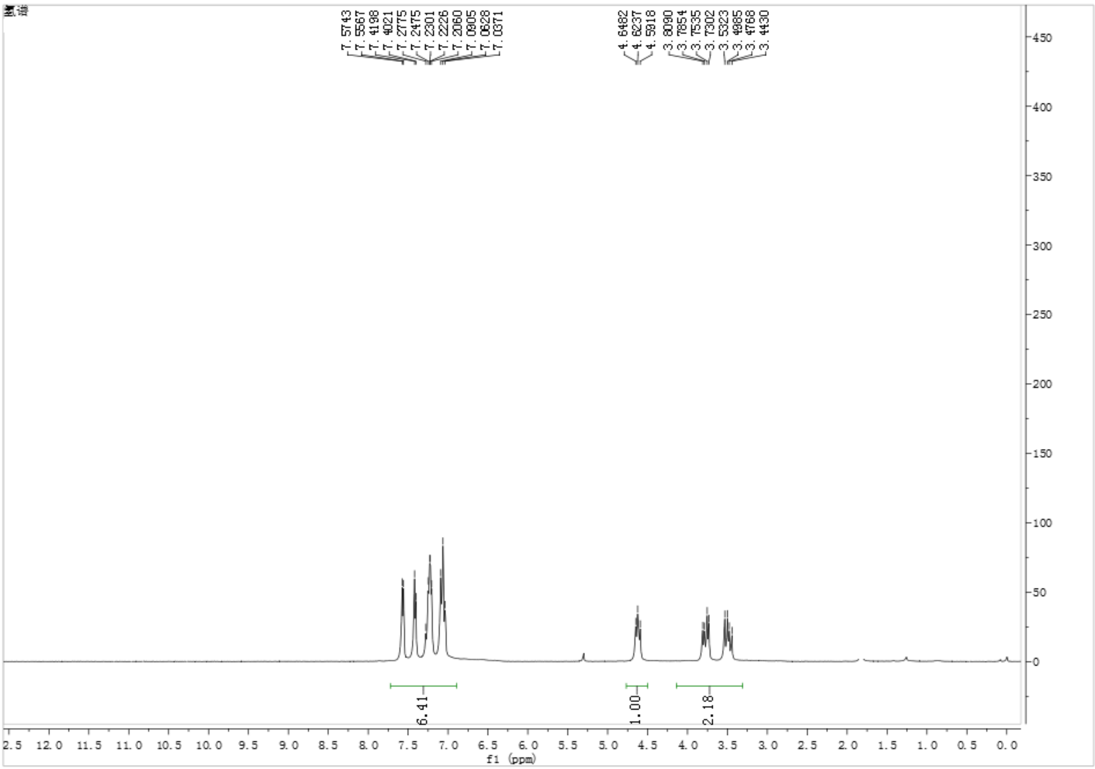

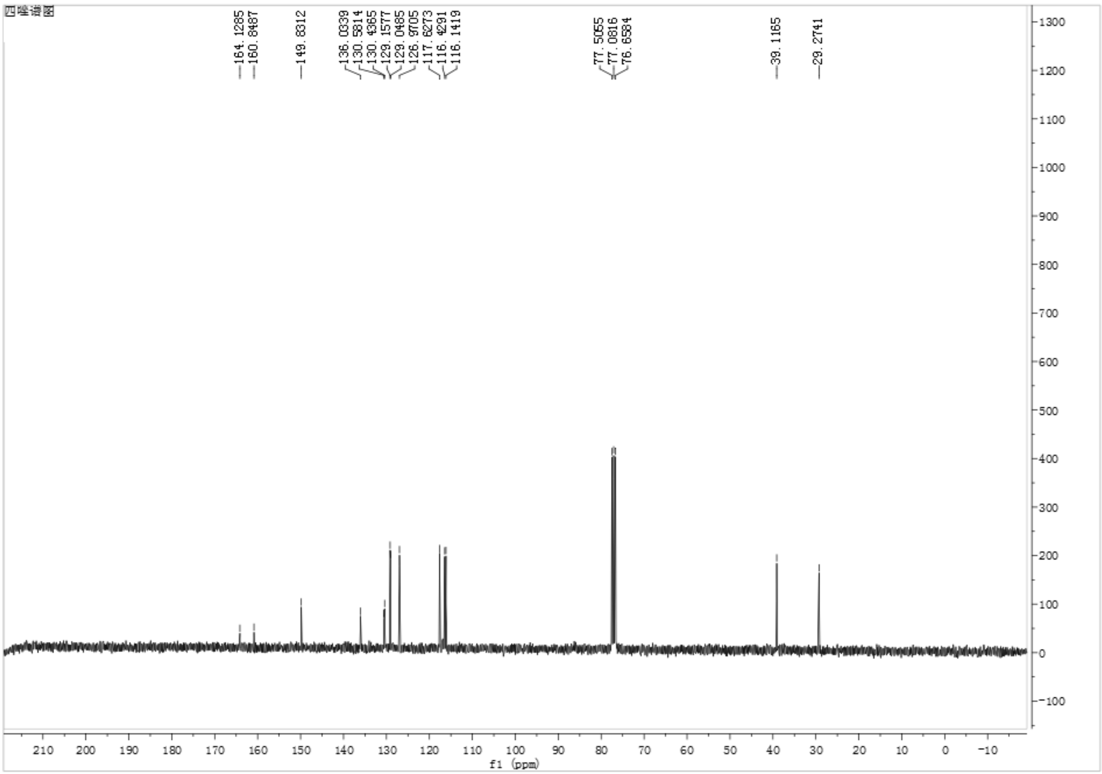

5-[2-(Trifluoromethyl)phenyl]-4,5-dihydrotetrazolo[1,5-a]thieno[2,3-e]pyridine (**4g**)

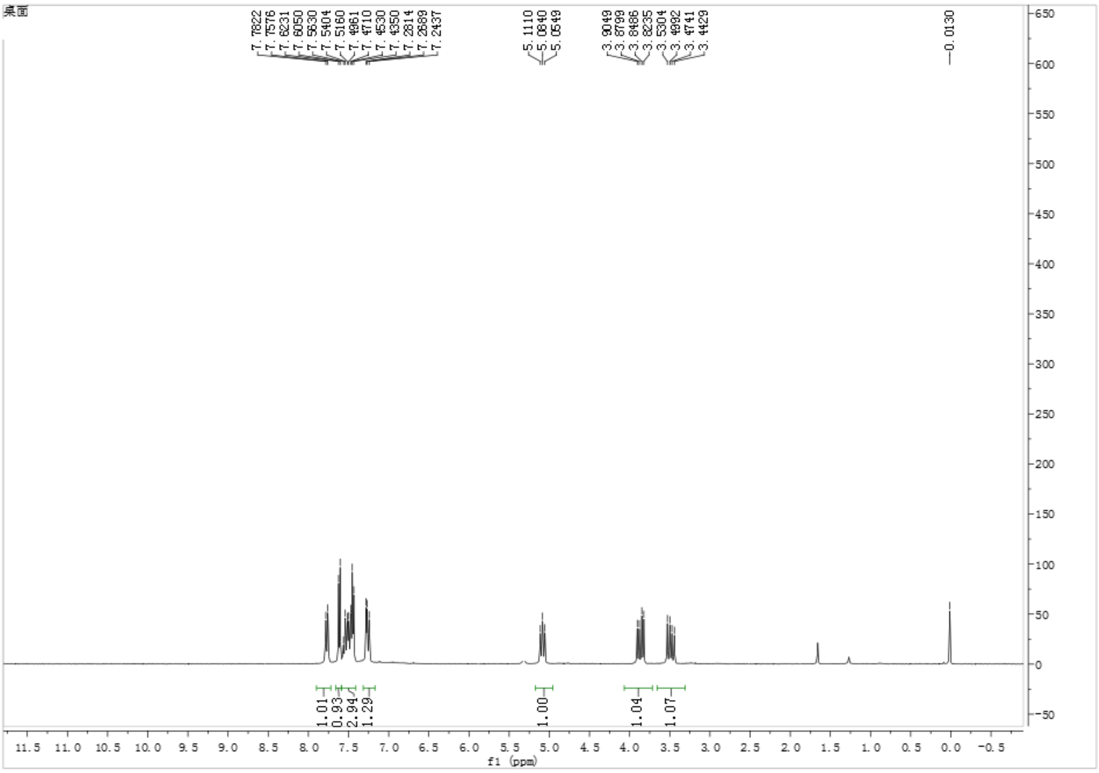

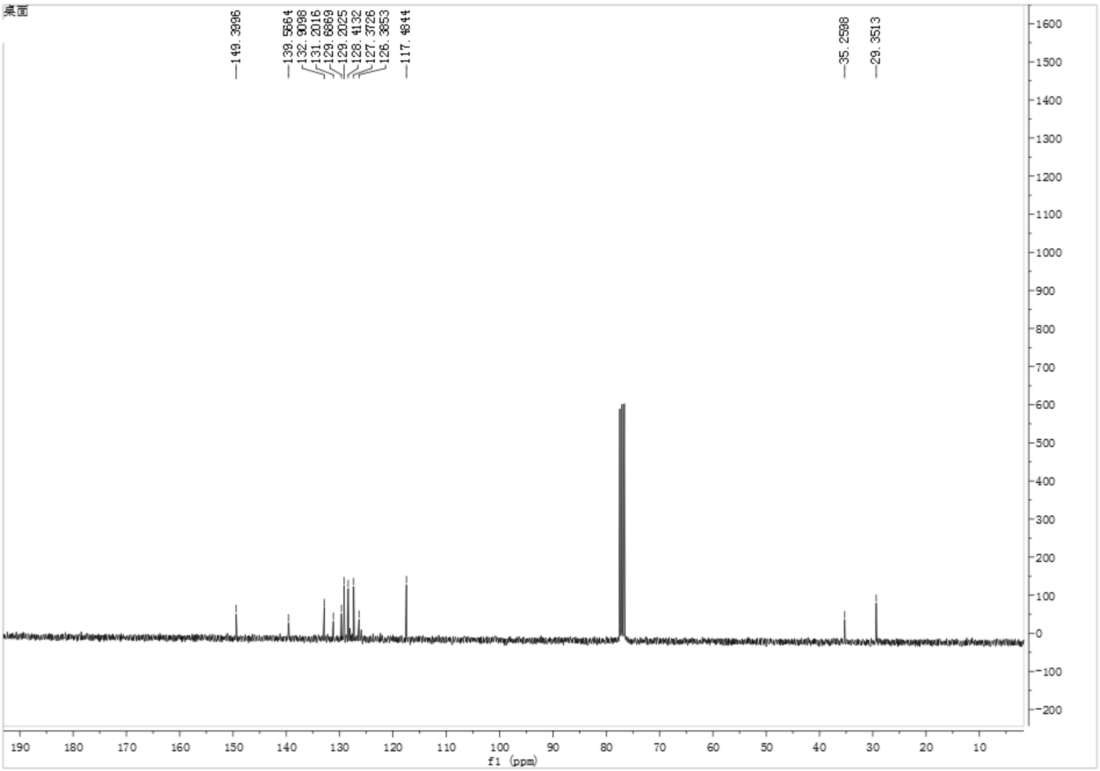

5-[3-(Trifluoromethyl)phenyl]-4,5-dihydrotetrazolo[1,5-a]thieno[2,3-e]pyridine (**4h**)

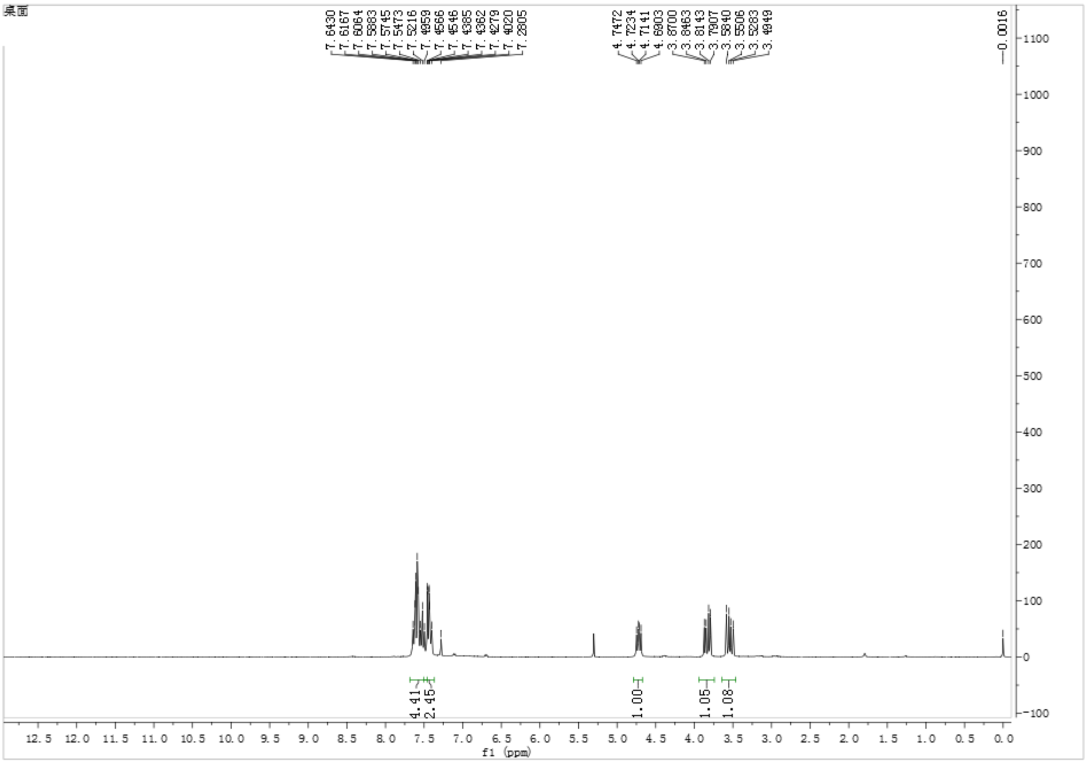

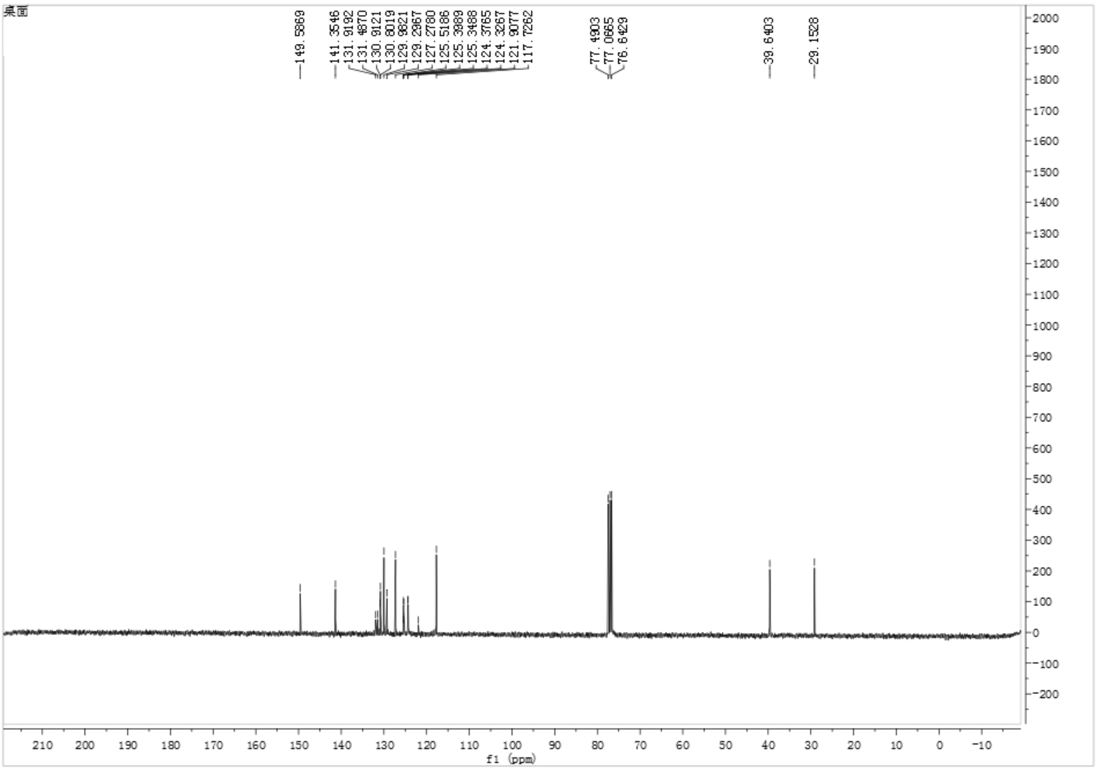

5-[4-(Trifluoromethyl)phenyl]-4,5-dihydrotetrazolo[1,5-a]thieno[2,3-e]pyridine (**4i**)

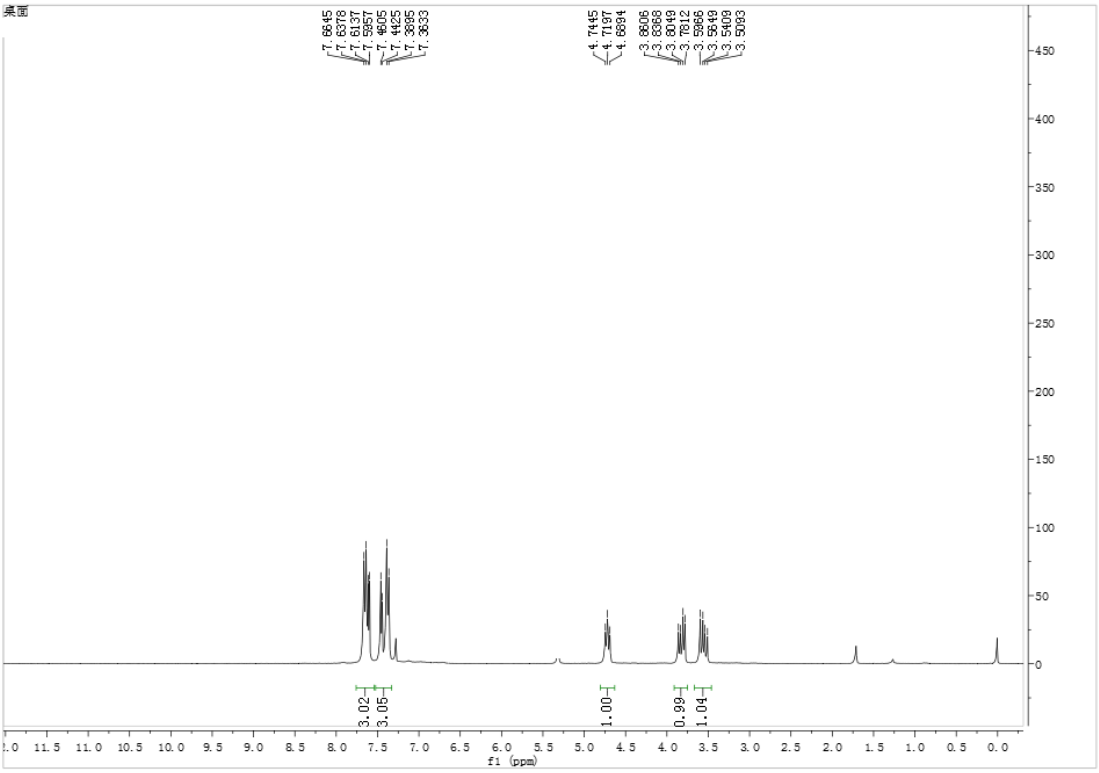

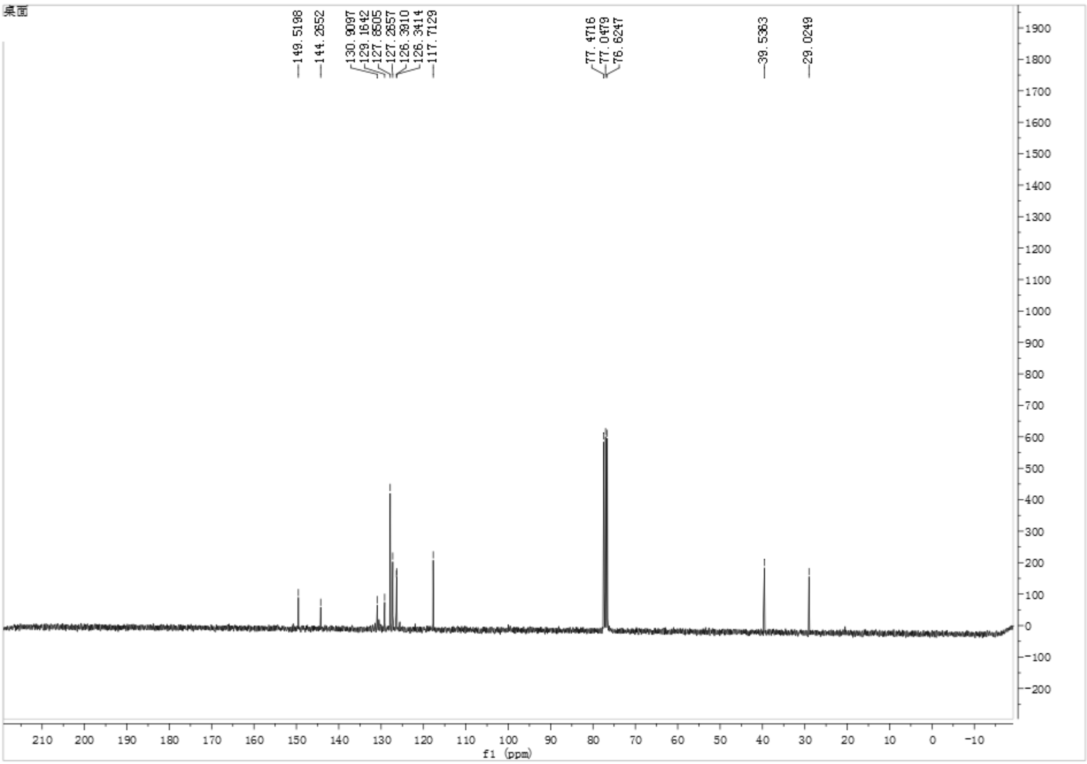

5-(2-Methoxyphenyl)-4,5-dihydrotetrazolo[1,5-a]thieno[2,3-e]pyridine (**4j**)

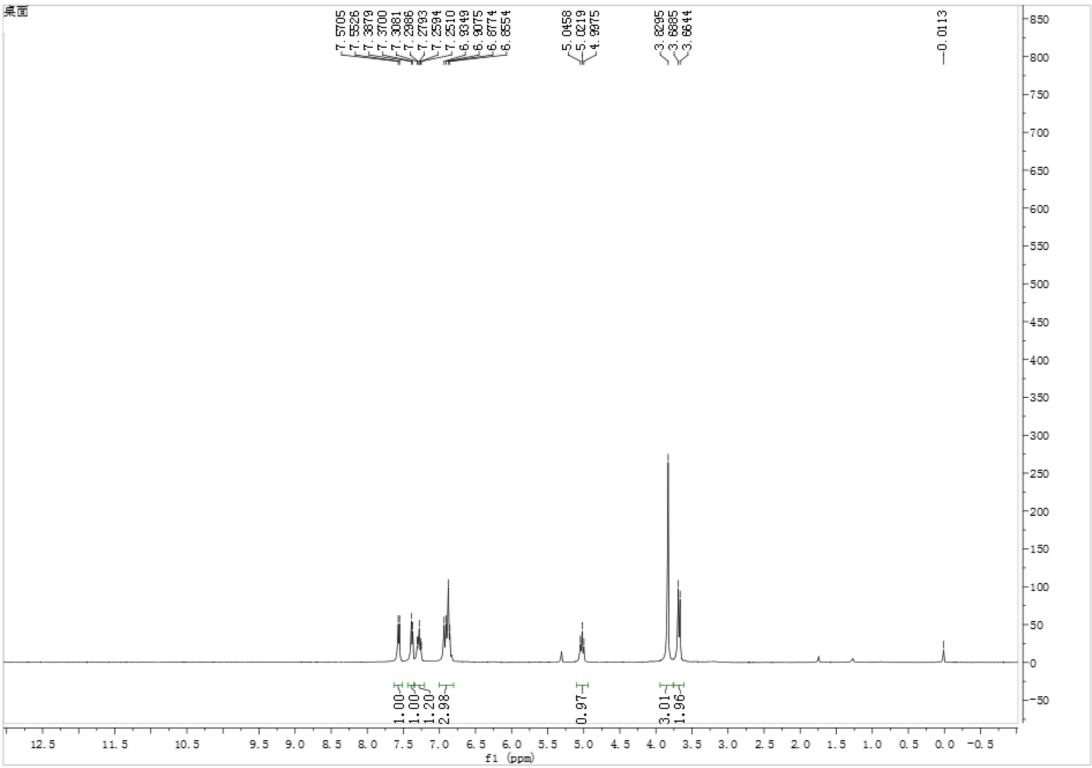

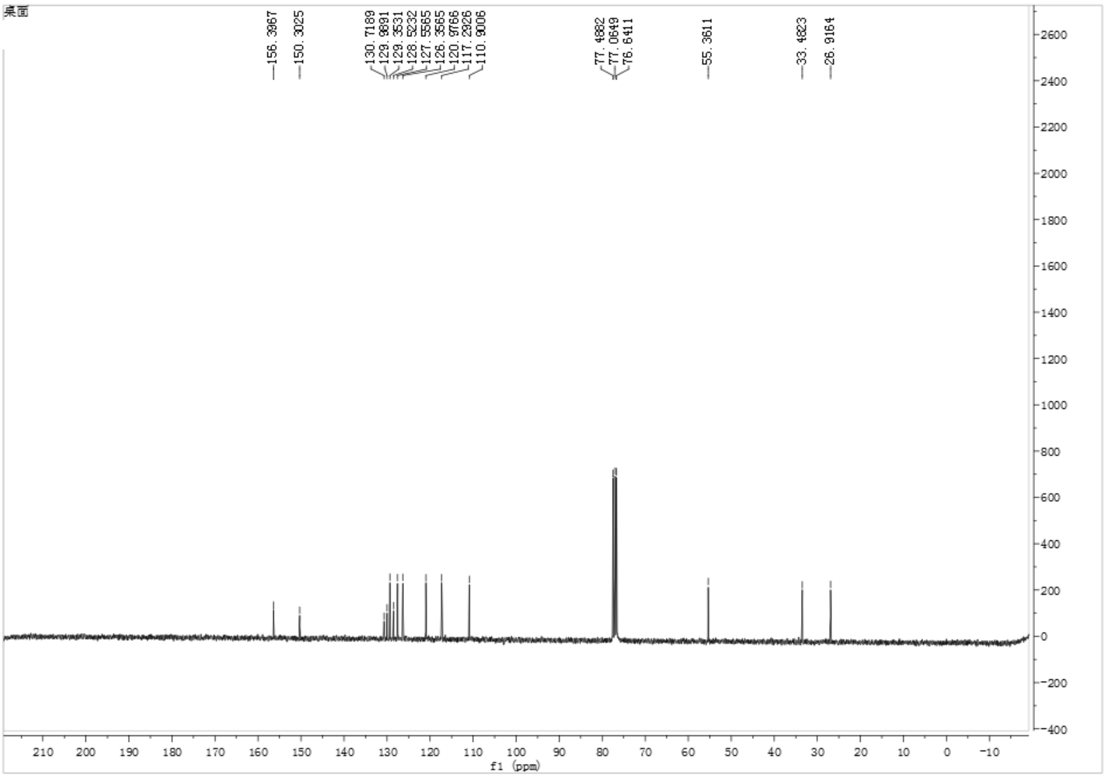

5-(3-Methoxyphenyl)-4,5-dihydrotetrazolo[1,5-a]thieno[2,3-e]pyridine (**4k**)

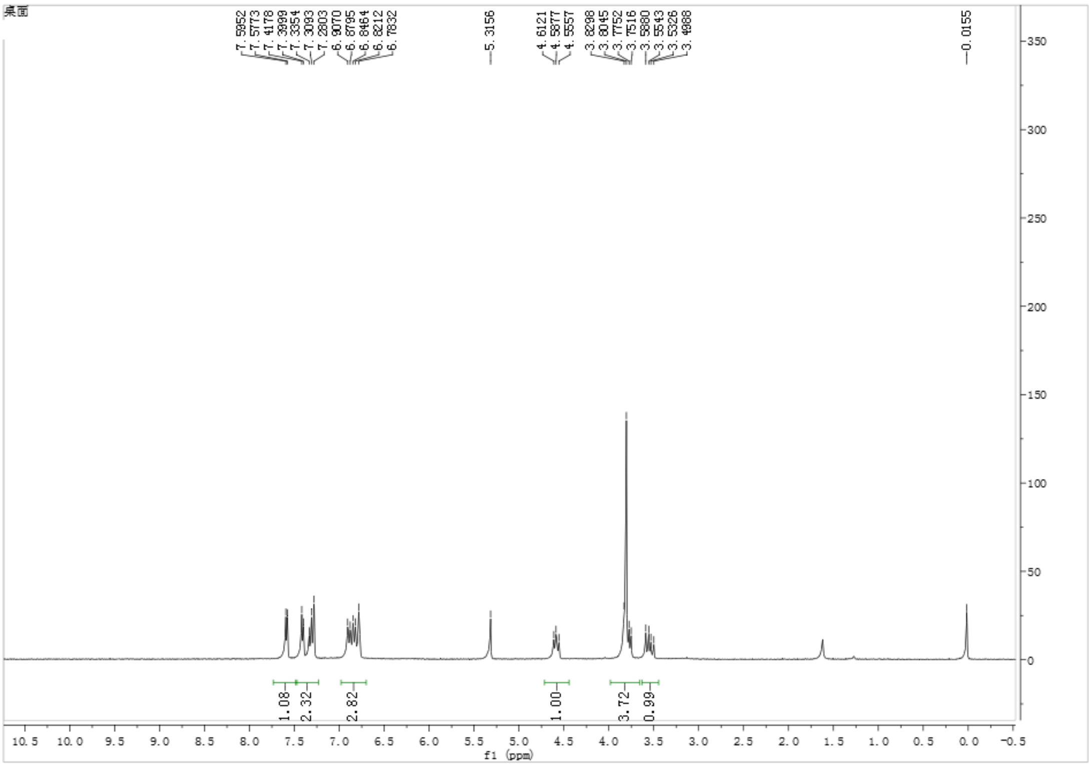

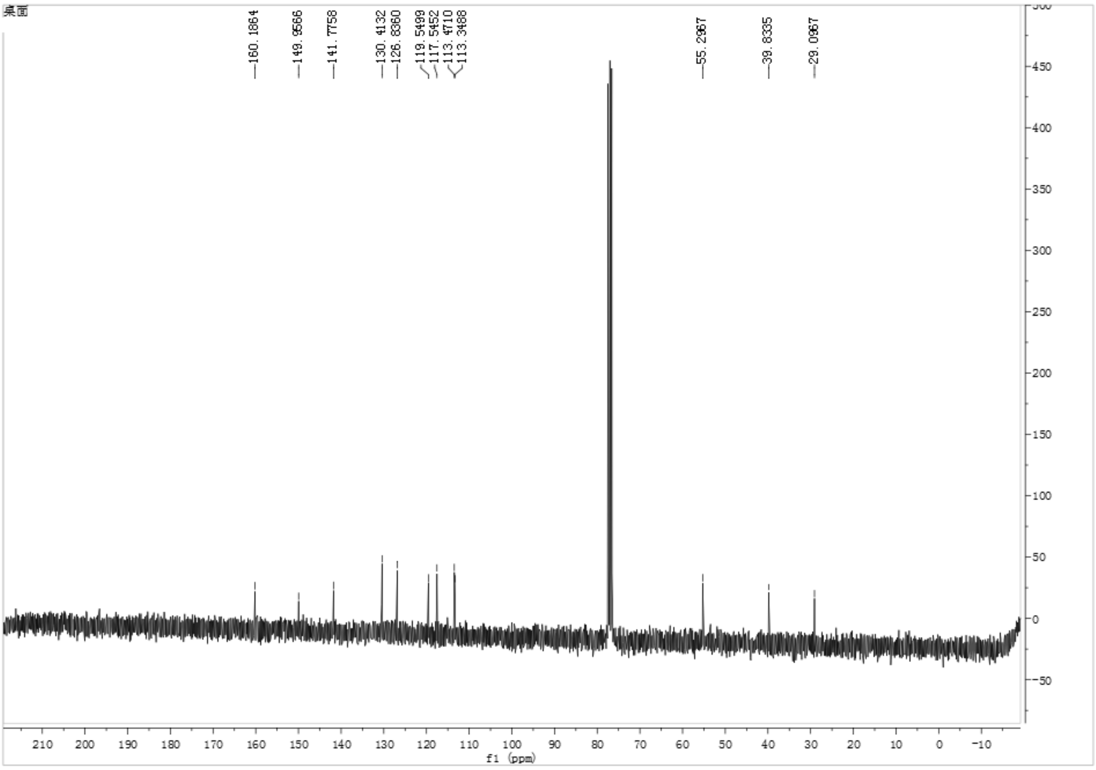

5-(4-Methoxyphenyl)-4,5-dihydrotetrazolo[1,5-a]thieno[2,3-e]pyridine (**4l**)

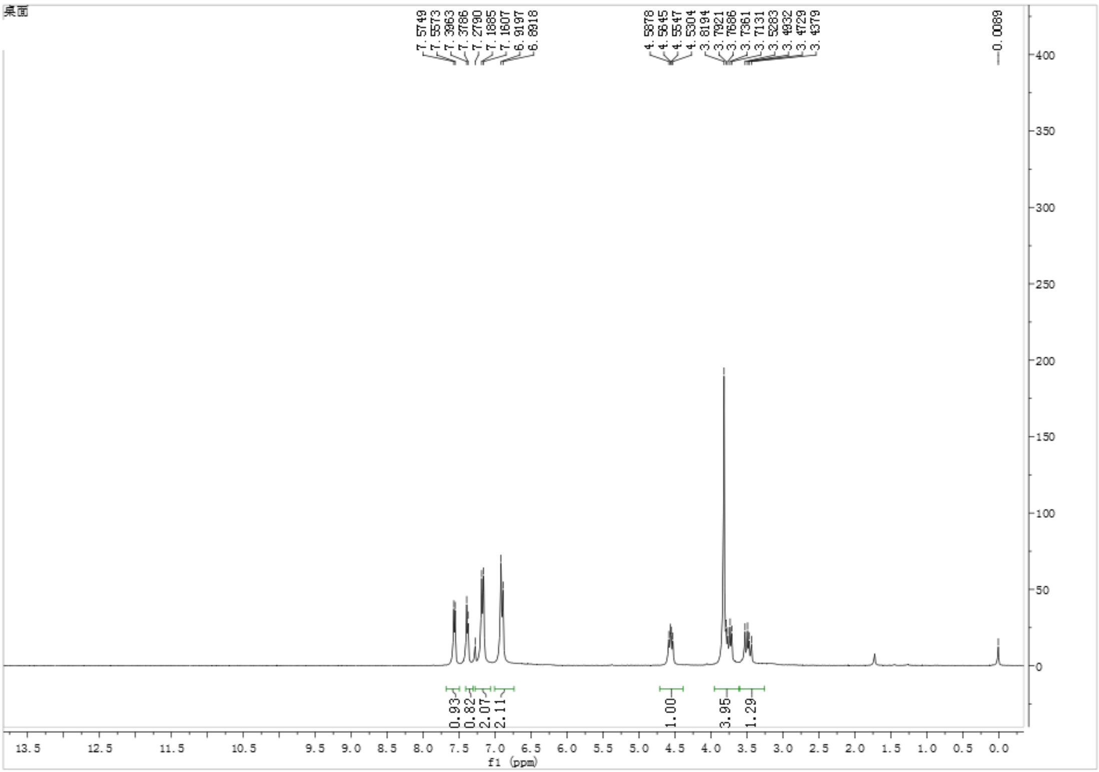

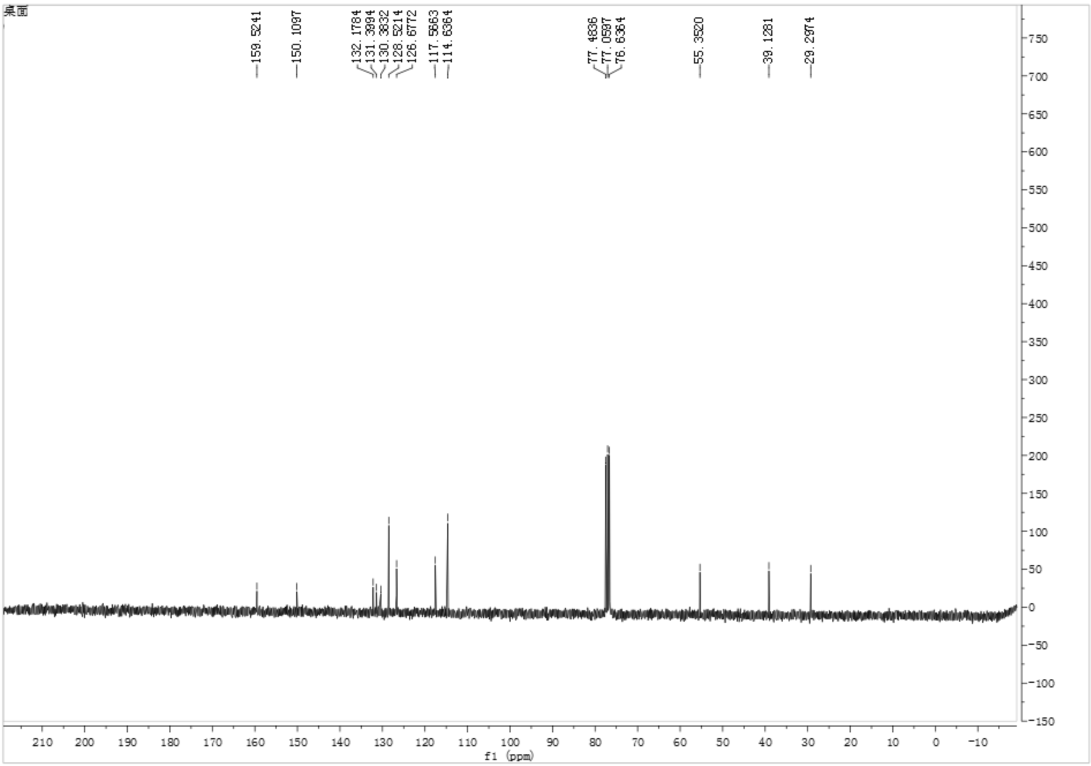

5-Phenyl-4,5-dihydrotetrazolo[1,5-a]thieno[2,3-e]pyridine(**4n**)

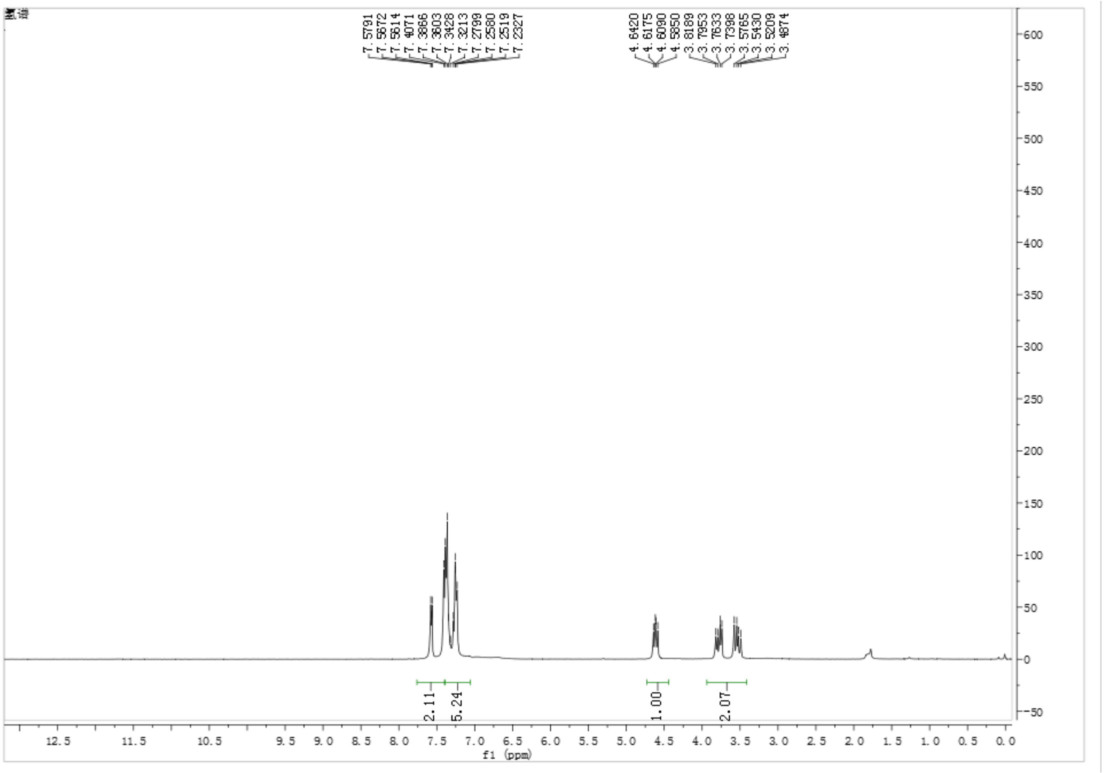

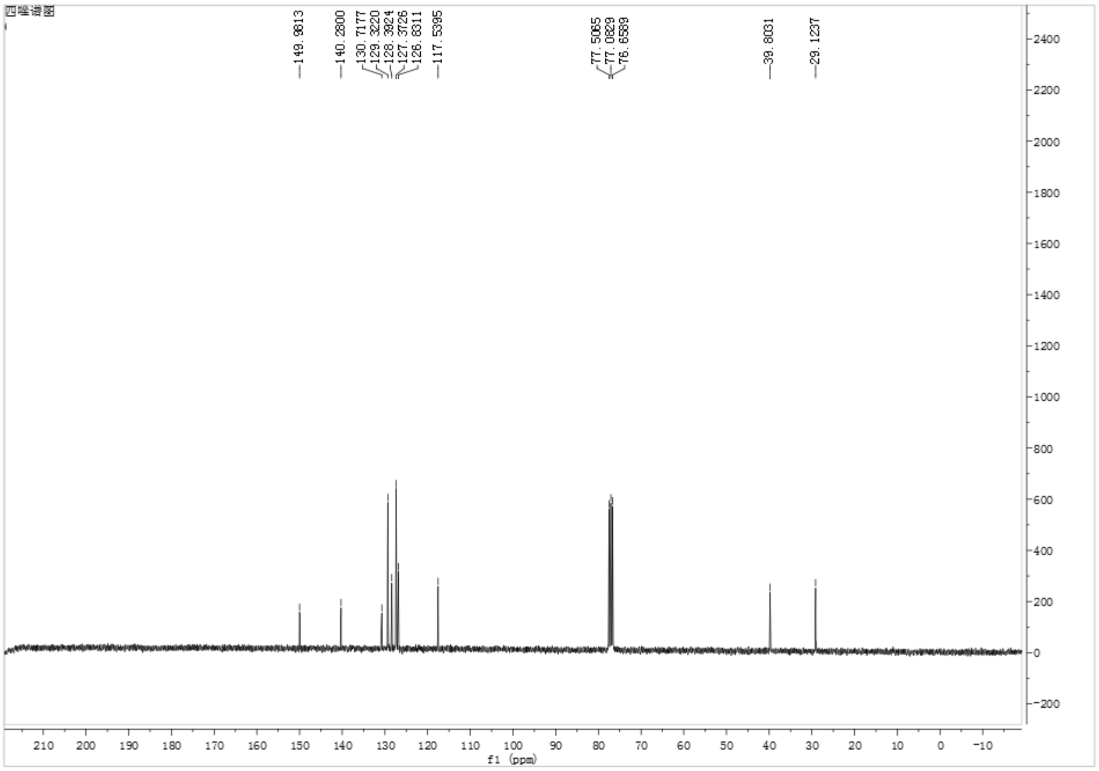

5-(2-Chloro-6-fluorophenyl)-4,5-dihydrotetrazolo[1,5-a]thieno[2,3-e]pyridine**(4o)**

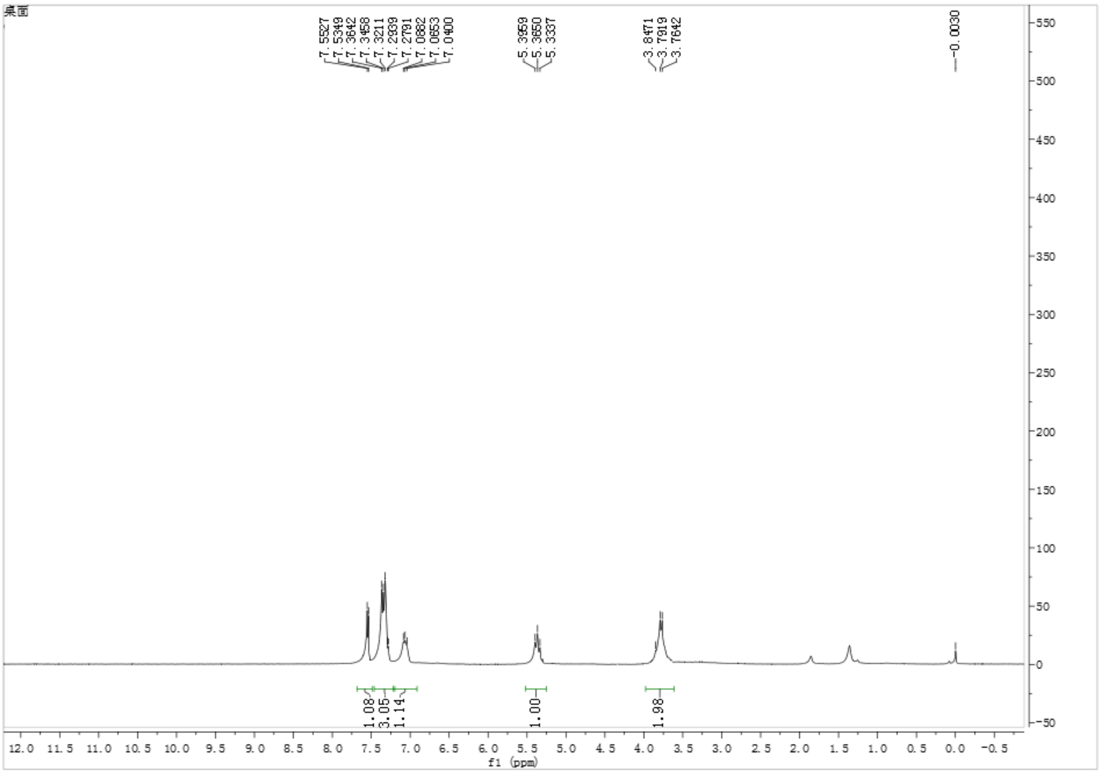

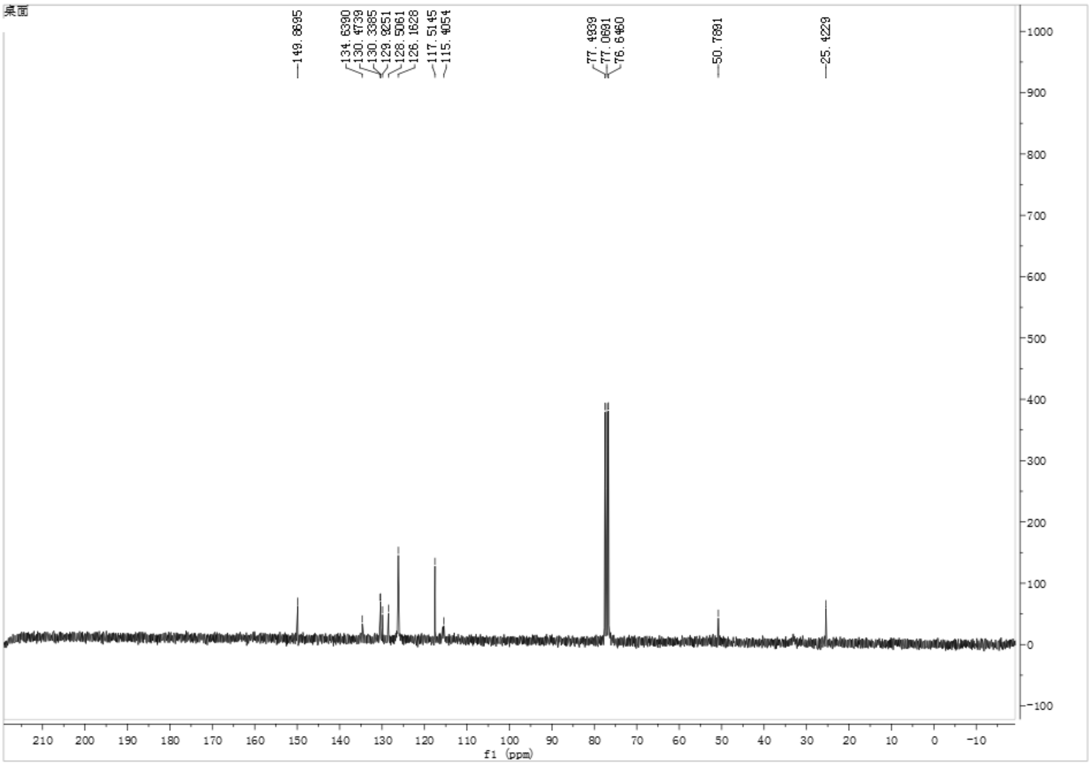

5-(3,4,5-Trimethoxyphenyl)-4,5-dihydrotetrazolo[1,5-a]thieno[2,3-e]pyridine (**4p**)

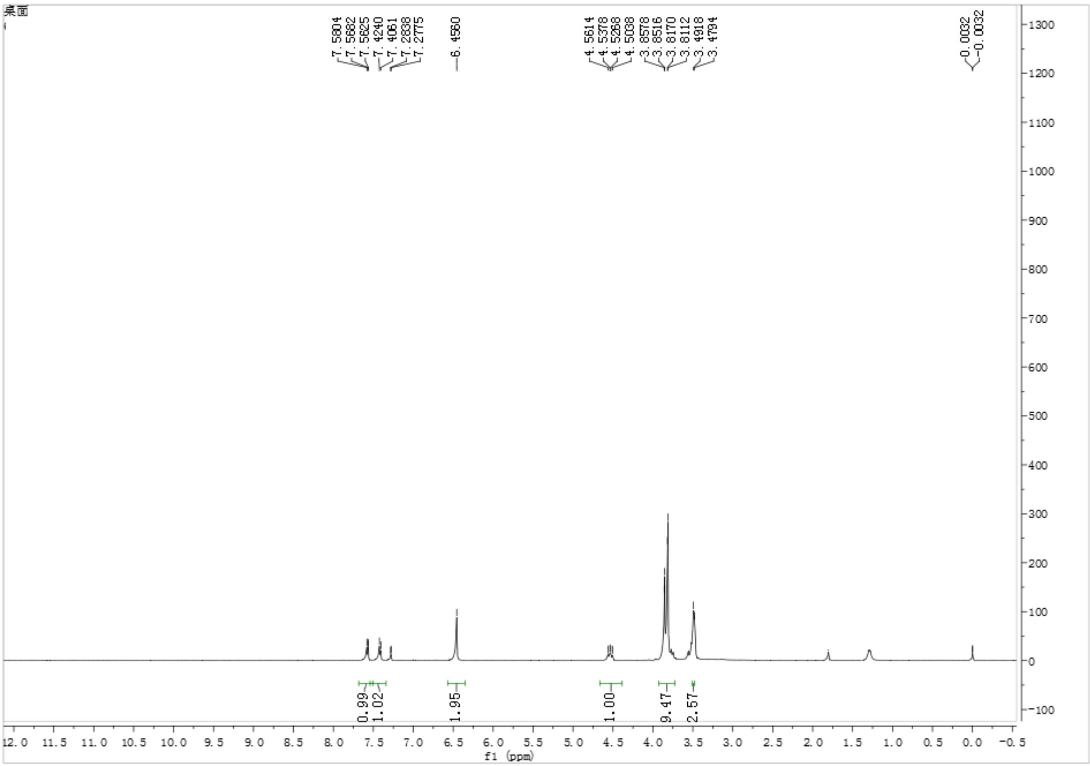

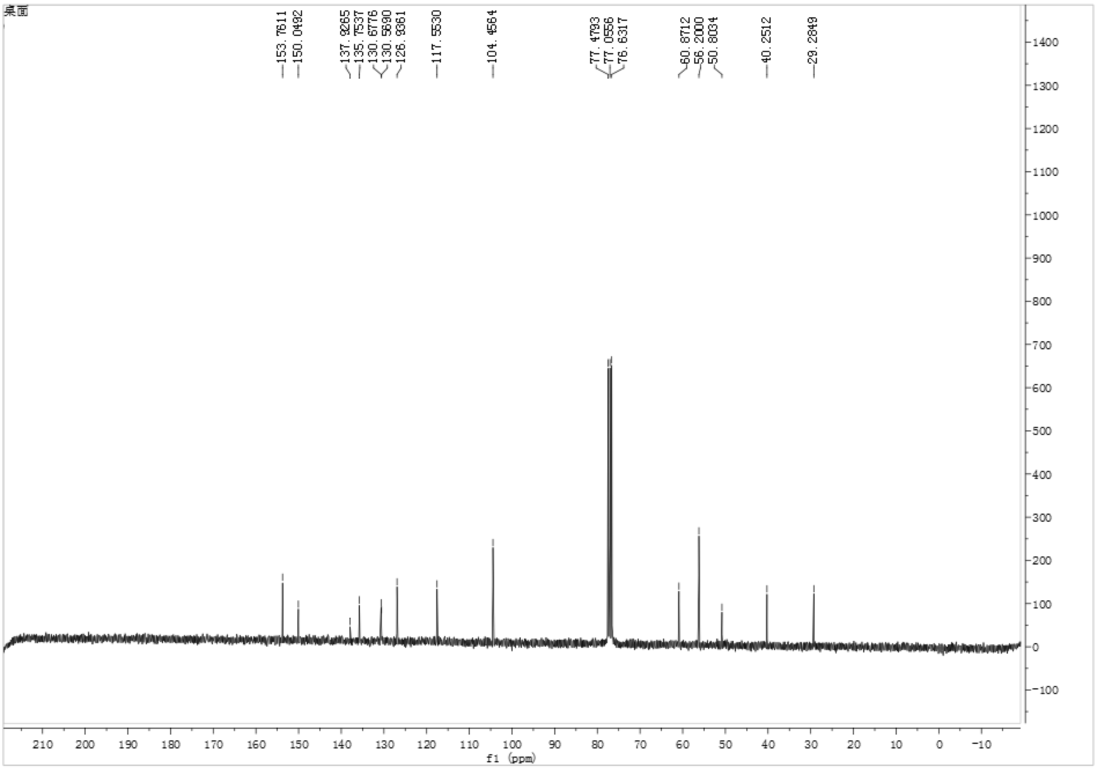

5-[4-(Trifluoromethyl)phenyl]-4,5-dihydrothieno[2,3-e][1,2,4]triazolo[4,3-a]pyridine (**5**)

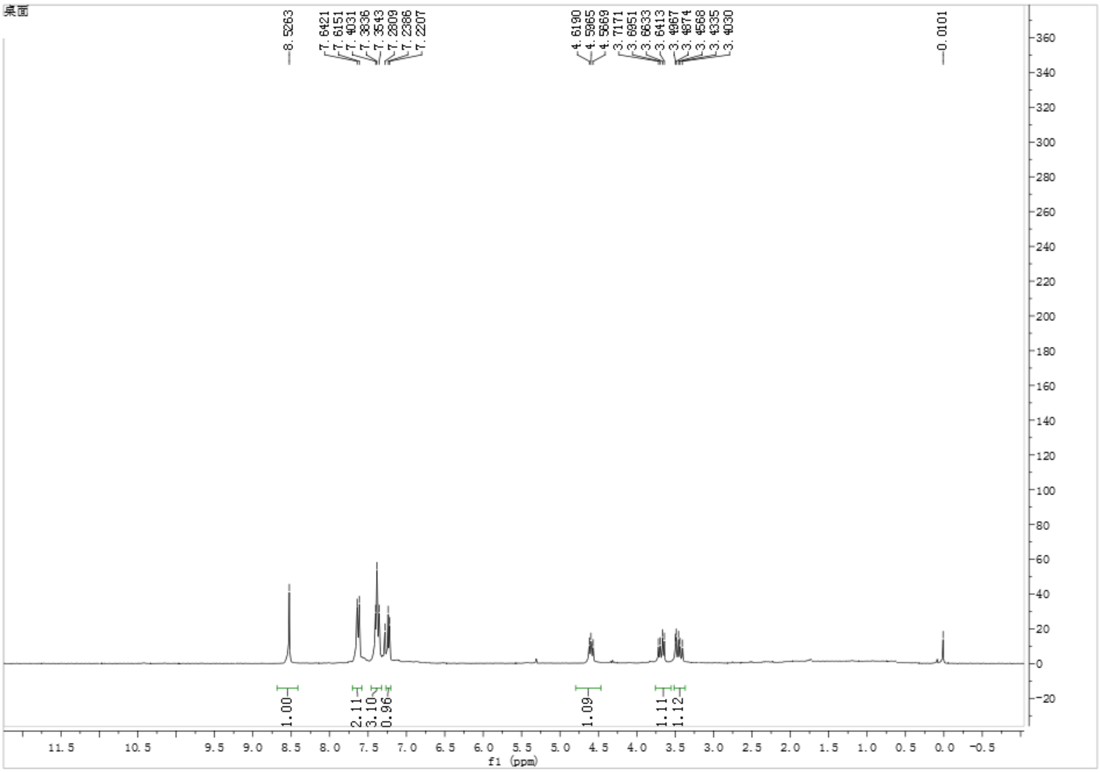

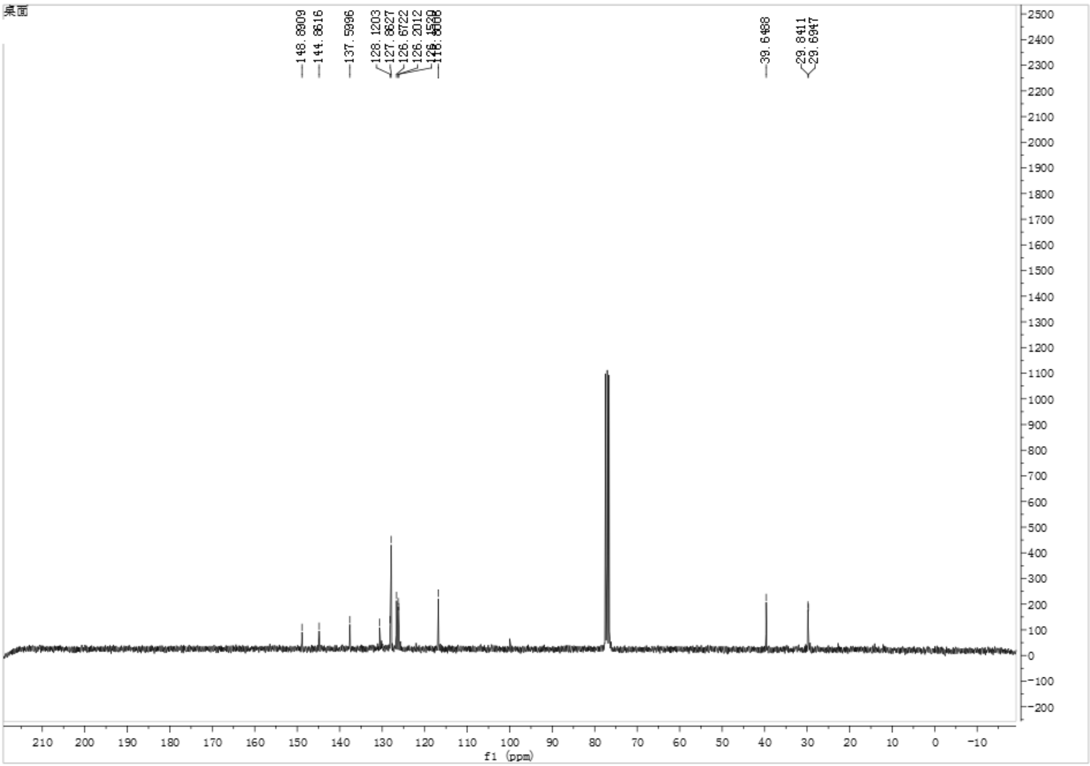

1-Methyl-5-[4-(trifluoromethyl)phenyl]-4,5-dihydrothieno[2,3-e][1,2,4]triazolo[4,3-a]pyridine (**6**)

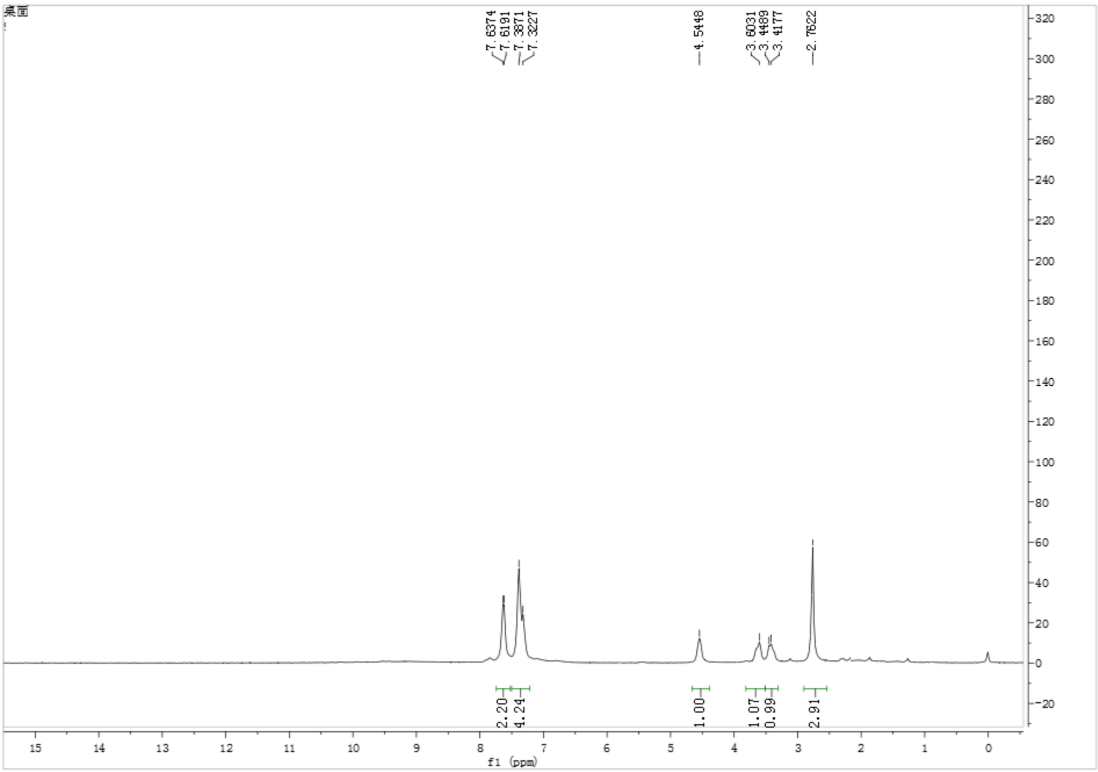

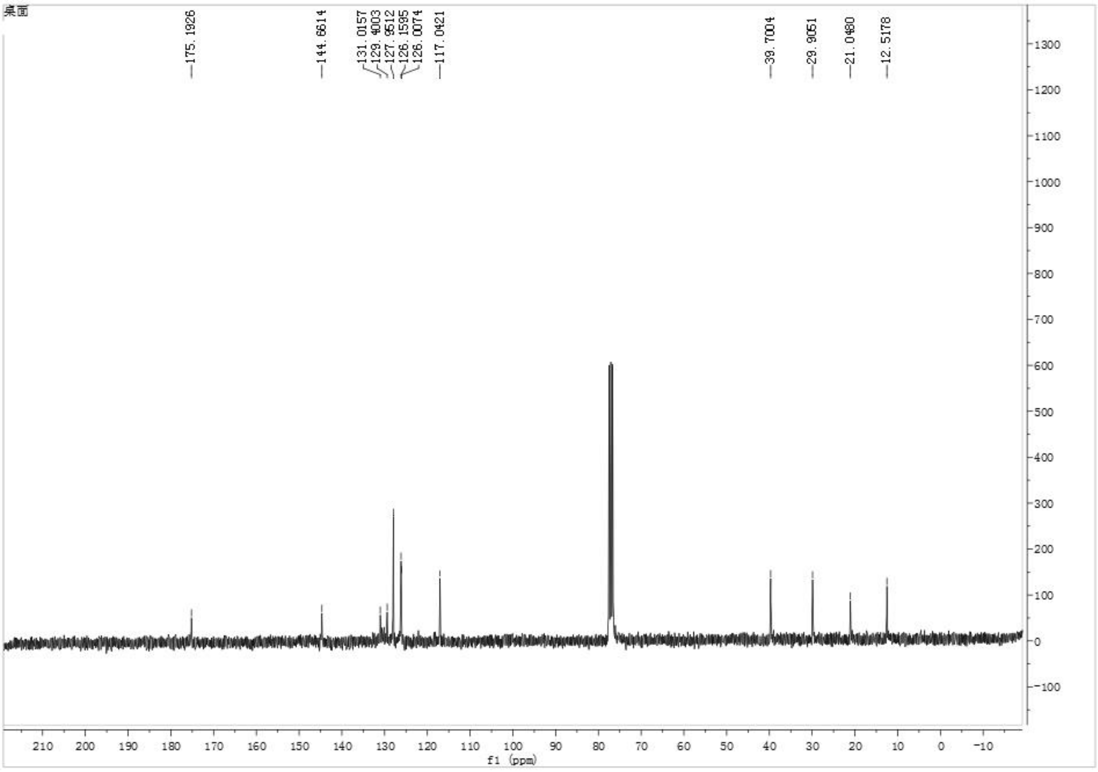

5-[4-(Trifluoromethyl)phenyl]-4,5-dihydrothieno[2,3-e][1,2,4]triazolo[4,3-a]pyridin-1(2H)-one (**7**)

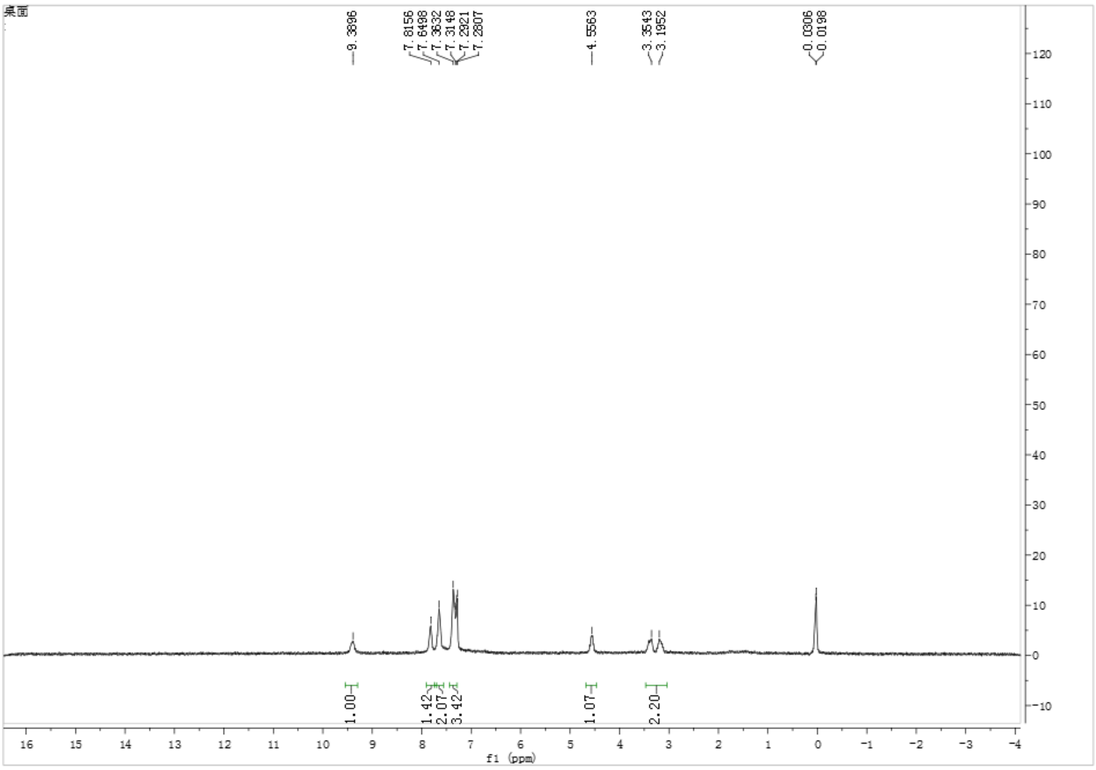

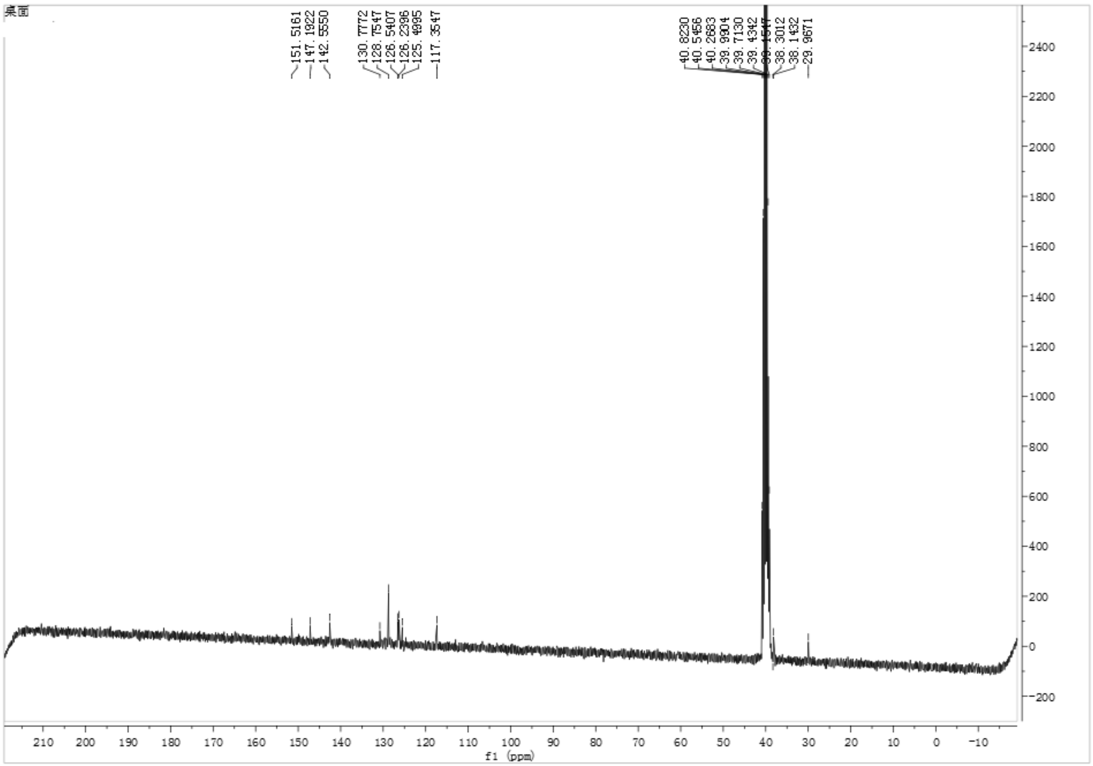

Supplement: Supplementary file 1 [file molecules-24-01857-s001.pdf]
